# Integrative Computational Approaches for the Discovery of Triazole-Based Urease Inhibitors: A Machine Learning, Virtual Screening, and Meta-Dynamics Framework

**DOI:** 10.3390/ijms262311576

**Published:** 2025-11-28

**Authors:** Sofía E. Ríos-Rozas, Natalia Morales, Elizabeth Valdés-Muñoz, Gabriela Urra, Camila A. Flores-Morales, Javier Farías-Abarca, Erix W. Hernández-Rodríguez, Jonathan M. Palma, Manuel I. Osorio, Osvaldo Yáñez-Osses, Luis Morales-Quintana, Reynier Suardíaz, Daniel Bustos

**Affiliations:** 1 Laboratorio de Bioinformática y Química Computacional, Departamento de Medicina Traslacional, Facultad de Medicina, Universidad Católica del Maule, Talca 3480094, Chilejavier.farias@alu.ucm.cl (J.F.-A.); ehernandez@ucm.cl (E.W.H.-R.); 2 Doctorado en Ingeniería, Facultad de Ingeniería, Universidad Católica del Maule, Talca 3480094, Chile; 3 Doctorado en Biotecnología Traslacional, Facultad de Ciencias Agrarias y Forestales, Universidad Católica del Maule, Talca 3480094, Chile; 4 Facultad de Ingeniería, Universidad de Talca, Maule, Curicó 3349001, Chile; jonathan.palma@utalca.cl; 5 Facultad de Odontología, Universidad Andrés Bello, Santiago Chile, Echaurren 237, Santiago 8370133, Chile; manuel.osorio@unab.cl; 6 Centro de Modelación Ambiental y Dinámica de Sistemas (CEMADIS), Facultad de Ingeniería y Negocios, Universidad de Las Américas, Santiago 7500975, Chile; oyanez@udla.cl; 7 Multidisciplinary Agroindustry Research Laboratory, Instituto de Ciencias Biomédicas, Facultad de Ciencias de La Salud, Universidad Autónoma de Chile, Cinco Poniente #1670, Región del Maule, Talca 7500912, Chile; luis.morales@uautonoma.cl; 8 Departamento de Química Física, Facultad de Ciencias Químicas, Universidad Complutense de Madrid, 28040 Madrid, Spain

**Keywords:** virtual screening, machine learning, quantum-polarized ligand docking, metadynamics, urease

## Abstract

*Helicobacter pylori* urease (*Hp*U) plays a central role in bacterial survival and virulence by hydrolyzing urea into ammonia and carbon dioxide, neutralizing gastric acidity, and facilitating host colonization. The increasing prevalence of antibiotic resistance underscores the need for alternative strategies targeting essential bacterial enzymes such as urease. In this study, a multistage computational pipeline integrating pharmacophore modeling, machine learning (ML), ensemble docking, and enhanced molecular dynamics simulations were applied to identify novel triazole-based *Hp*U inhibitors. Starting from over seven million compounds in the ZINC15 database, pharmacophore- and ML-based filters progressively reduced the chemical space to 7062 candidates. Ensemble docking across 25 conformational frames of *Hp*U, followed by quantum-polarized ligand docking (QPLD), identified seven promising ligands exhibiting strong binding energies and stable metal coordination. Molecular dynamics (MD) simulations under progressively relaxed restraints revealed three highly stable complexes (CA1, CA3, and CA6). Subsequent well-tempered metadynamics (WT-MetaD) simulations reconstructed free-energy landscapes showing deep, localized basins for CA3 and CA6, comparable to the potent reference inhibitor DJM, supporting their potential as strong urease binders. Finally, unsupervised chemical space mapping using the UMAP algorithm positioned these candidates within molecular regions associated with potent urease inhibitors, further validating their structural coherence and pharmacophoric relevance. An ADMET assessment confirmed that the selected candidates exhibit physicochemical and early safety properties compatible with subsequent in vitro evaluation. This multilevel screening strategy demonstrates the power of combining ML-driven classification, ensemble docking, and enhanced sampling simulations to discover non-hydroxamic urease inhibitors. Although the current findings are computational, they provide a rational foundation for future in vitro validation and for expanding the discovery of triazole-based scaffolds targeting ureolytic enzymes.

## 1. Introduction

*Helicobacter pylori* (*Hp*) infection remains a major global health burden, affecting nearly half of the world’s population [1]. It is the primary etiological agent of chronic gastritis, peptic ulcers, and gastric cancer [2]. Emerging evidence also implicates *Hp* in extra-digestive disorders through its modulation of the gut–brain–microbiota axis [3]. *Hp* has been classified as a Group I carcinogen by the World Health Organization [4], underscoring the need for more effective therapeutic strategies.

Among its virulence factors, urease is central to *Hp* colonization and persistence in the acidic gastric niche. This nickel-dependent metalloenzyme hydrolyzes urea into ammonia and carbon dioxide, buffering the microenvironment and enabling bacterial survival [5]. The resulting ammonia contributes to epithelial damage and inflammation [6]. Current pharmacological regimens combining antibiotics and proton-pump inhibitors are losing efficacy due to increasing antimicrobial resistance [7], highlighting the importance of alternative approaches that target essential bacterial functions such as urease activity [8].

This epidemiological landscape has intensified scientific interest in the development of specific urease inhibitors (UI) as alternative or complementary strategies for controlling *Hp* infection. By targeting a mechanism essential for bacterial survival, UI offer a therapeutic advantage since they can disrupt colonization without exerting the selective pressure typically associated with conventional antibiotics, thereby reducing the likelihood of resistance development [9,10]. Triazole-based inhibitors are of particular interest due to their favorable physicochemical properties, including heteroaromatic nitrogen atoms, well-defined hydrogen-bonding patterns, and rigid scaffolds, that contribute to enhanced anti-urease activity [11,12]. Several triazole derivatives have demonstrated potent inhibition of *Hp* urease (*Hp*U) and may reduce the likelihood of resistance development [13].

The paradigm of drug discovery has undergone a profound transformation with the integration of computational methodologies. These tools have revolutionized the identification and optimization of bioactive compounds, streamlining the discovery process by significantly lowering both time and cost compared to traditional experimental approaches [14,15]. Structural bioinformatics enables the detailed molecular characterization of *Hp* urease (*HpU*), including the analysis of its three-dimensional conformation, catalytic mechanisms, and potential binding pockets. Molecular docking enables the virtual screening (VS) of extensive chemical libraries by generating plausible ligand–protein binding poses and providing scoring functions that approximate relative interaction strength, rather than true binding affinities [16]. Similarly, molecular dynamics simulations provide insight into the stability of enzyme–inhibitor complexes under physiological conditions and the mechanisms of inhibition [17,18].

Machine learning (ML) algorithms complement these physics-based approaches by identifying non-obvious structure–activity relationships within large datasets and predicting pharmacological properties relevant to urease inhibition [19,20,21,22]. Supervised learning classifiers, such as support vector machines (SVM), artificial neural networks, and random forests (RF), have shown remarkable efficacy in identifying molecular features associated with urease inhibitory activity [22]. The convergence of structural bioinformatics and ML methods offers a comprehensive framework for the rational search for UI, maximizing the likelihood of success in subsequent experimental phases [23,24].

**Figure 1:**
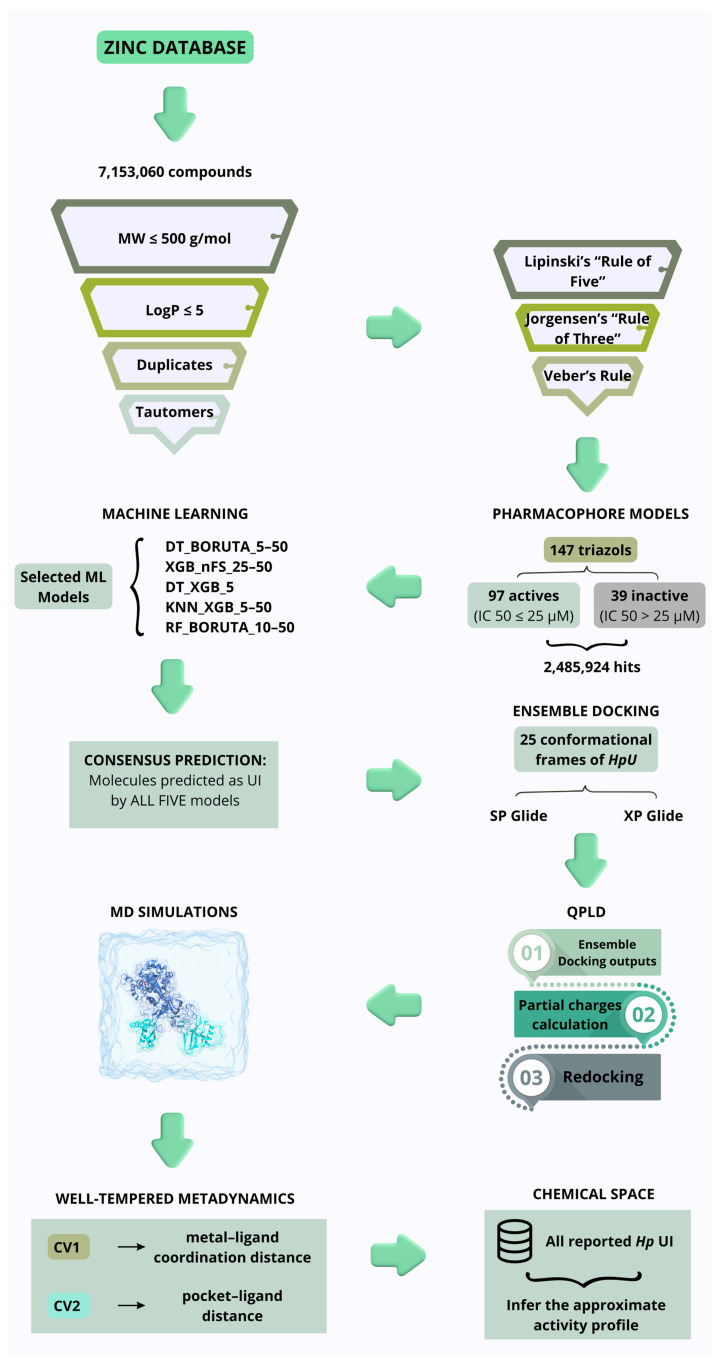
Schematic representation of the multistage computational workflow for the identification of novel triazole-based *Helicobacter pylori* urease (*Hp*U) inhibitors. The pipeline begins with the curation of compounds from the ZINC database, filtered by molecular weight, partition coefficient, and the removal of duplicates and tautomers. Subsequent pharmacokinetic screening applied Lipinski’s “Rule of Five,” Jorgensen’s “Rule of Three,” and Veber’s Rule. Pharmacophore models were then generated from 147 triazole-derived inhibitors, yielding 2,485,924 hits. Five machine learning (ML) classifiers were used to predict urease inhibitors (UI), and only molecules consistently predicted as active by all models were retained. These compounds underwent ensemble docking against 25 conformational frames of *Hp*U using SP and XP Glide protocols. The best-ranked ligands were refined through Quantum-Polarized Ligand Docking (QPLD), incorporating QM-derived partial charges. Molecular dynamics (MD) simulations were conducted to assess complex stability, followed by well-tempered metadynamics (WT-MetaD) using two collective variables (CV1: metal–ligand coordination distance; CV2: pocket–ligand distance) to explore the free-energy landscape. Finally, a chemical space analysis compared the top candidates with all reported *Hp*U inhibitors to infer their relative activity and structural coherence within the inhibitor landscape.

In this work, we present a multi-stage computational pipeline to identify novel triazole-based *Hp*U inhibitors (Figure 1). Beginning with the ZINC database, compounds were filtered according to physicochemical and drug-likeness criteria, then prioritized using a triazole-derived pharmacophore model and previously validated ML classifiers [20]. Promising candidates underwent sequential structure-based evaluation, including ensemble docking across 25 conformational states of *Hp*U, quantum-polarized ligand docking (QPLD), all-atom MD simulations, and well-tempered metadynamics (WT-MetaD). To contextualize their predicted activity, the selected ligands were additionally mapped within the reported UI chemical space extracted from ChEMBL, BindingDB, and PubChem. Finally, we assessed their pharmacokinetic and early-stage safety profiles using ADMET predictions to ensure that the top candidates would be viable for future in vitro validation. This integrated approach expands the repertoire of triazole-derived urease inhibitors and provides a rigorous, mechanistically grounded framework for prioritizing compounds with translational potential.

## 2. Results

### 2.1. Preliminary Database Curation and Physicochemical Filtering

The initial stage of this VS reproduced the same data curation and filtering pipeline previously validated by our group for the identification of *Hp* UI [14]. Briefly, the commercially available subset of the ZINC15 database was downloaded in mol2 format, restricted to molecules with molecular weight (MW) ≤ 500 g/mol and partition coefficient (LogP) ≤ 5. The initial library comprised 7,153,060 compounds. After the removal of redundant tautomers, representing approximately 7.5% of entries, the dataset was reduced to 6.6 million unique molecules. LigPrep (Schrödinger Release 2023-3) was used to generate up to two stereochemically plausible conformations per molecule. Subsequent filtering was conducted according to the pharmacokinetic criteria described in our previous work, based on Lipinski’s Rule of Five, Jorgensen’s Rule of Three, and the Veber Rule. This sequential filtering eliminated compounds violating thresholds for solubility, permeability, MW, hydrogen-bonding capacity, rotatable bonds, and polar surface area (PSA). The final curated dataset comprised 4,903,299 molecules, representing approximately 59% of the initially prepared library. As expected, these values were invariant compared with those reported in our prior study, given the use of identical databases and physicochemical criteria. This curated library served as the foundation for the subsequent pharmacophore-based and ML filtering stages.

### 2.2. Pharmacophore-Based VS (PBVS) Filtering

**Figure 2:**
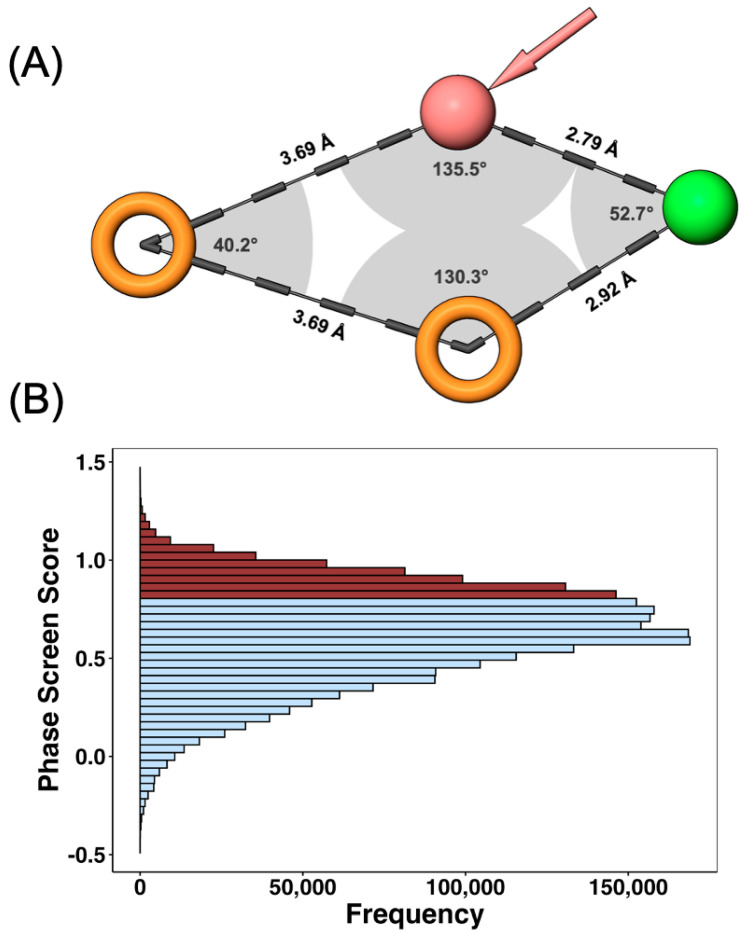
Pharmacophore model generation and screening of triazole-based urease inhibitors. (**A**) Representation of the optimal AHRR pharmacophore model highlighting the spatial arrangement of its four features: one hydrogen bond acceptor (A, red sphere), one hydrophobic group (H, green sphere), and two aromatic ring centers (R, orange rings). Distances and angular relationships between pharmacophoric points are shown in ångströms and degrees, respectively. (**B**) Frequency distribution of the Phase Screen Scores (PSS) for all 4,903,299 molecules aligned to the pharmacophore model. Compounds scoring above the third quartile (Q3 = 0.797, shaded in burgundy) were selected for subsequent ML-based filtering.

Using the Phase tool (Schrödinger Release 2023-3), four pharmacophore hypotheses were generated based on a curated dataset of triazole derivatives with experimentally validated urease inhibitory activity. Each hypothesis consisted of different combinations of hydrogen bond acceptors (A), hydrophobic groups (H), and aromatic rings (R). The performance of the resulting pharmacophore models was evaluated according to three complementary criteria: (i) the number and diversity of pharmacophoric features, (ii) the BEDROC score, and (iii) the active/inactive compound ratio within the training set (Table S1 in Supplementary Information). Among the models generated, AHRR and AAAR exhibited the best discrimination metrics, with the Boltzmann-enhanced discrimination of receiver operating characteristic (BEDROC) scores of 0.627 and 0.622, respectively. Although AAAR displayed a slightly higher active/inactive ratio (35 versus 24.66), AHRR was ultimately selected as the optimal hypothesis due to its greater structural diversity and superior BEDROC score, which better reflects the overall enrichment capacity of the model. Figure 2A illustrates the spatial configuration and geometric relationships among the four pharmacophoric sites, highlighting the distances between 2.7 and 3.6 ångströms (Å) and angular orientations that define the active conformation. The BEDROC score is a quantitative metric that measures the early recognition capability of a pharmacophore model, providing a probability-based estimation (ranging from 0 to 1) of correctly identifying active compounds over random selection. High BEDROC values thus indicate stronger discriminatory power of the pharmacophore hypothesis.

The selected AHRR pharmacophore was then applied to the filtered ZINC15 library, comprising 4,903,299 molecules. Of these, 2,485,924 compounds matched the pharmacophoric hypothesis (Figure 2B). The distribution of Phase Screen Scores (PSS), which quantify the degree of alignment between each compound and the pharmacophore model, ranged from −0.489 to 1.434 (mean = 0.617 ± 0.244). Based on quartile analysis, the third quartile (Q3 = 0.797) was used as the selection threshold, retaining the top 623,350 molecules for subsequent ML filtering. These high-PSS molecules are predicted to most closely resemble the chemical and geometric characteristics of known triazole-based UI.

### 2.3. ML-Based VS Filtering

To refine the pharmacophore-enriched dataset, a multilayered ML filtering strategy was applied using the five classification models previously developed and validated by our group [20]. Each model: DT_BORUTA_5_50, XGB_nFS_25_50, DT_XGB_5, KNN_XGB_5_50, and RF_BORUTA_10_50, was independently used to predict the UI potential of the 623,350 compounds retained after the PBVS stage. It is important to note that the five ML models used here were previously trained and validated on a chemically diverse set of *Hp*U inhibitor, including triazoles, thiazoles, coumarins, and phenolic derivative, as reported in Morales et al. (2024) [20]. Thus, the classification stage in this study operates entirely within the established applicability domain of these models, which already includes the 147 triazole-derived inhibitors referenced in the literature.

**Figure 3:**
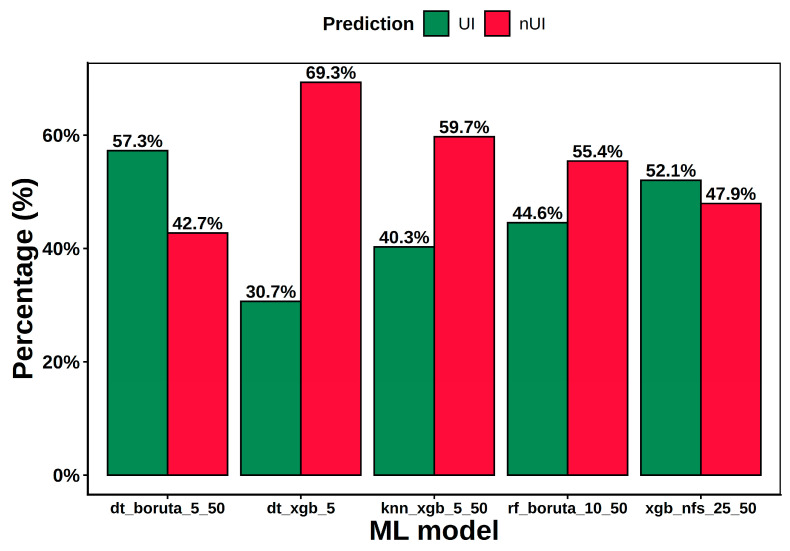
Comparative prediction outcomes of the five ML models applied to triazole-based UI candidates. Comparison of ML model predictions for the 623,350 compounds selected after PBVS. Green bars represent the percentage of molecules classified as UI, and red bars correspond to nUI. Each model applies a distinct algorithm, feature selection method, and bioactivity cutoff threshold, resulting in variable prediction distributions across models.

Figure 3 illustrates the comparative distribution of predictions across models, expressed as the percentage of molecules classified as UI (green bars) or non-UI (nUI, red bars). The observed differences among models reflect their distinct learning algorithms (e.g., DT_), feature selection strategies (e.g., BORUTA_), and bioactivity cutoff definitions (e.g., 5_50 μM). In detail, DT_BORUTA_5–50 was the most permissive model, classifying 57.3% of compounds as UI (42.7% nUI), while XGB_nFS_25–50 yielded a near-balanced outcome (52.1% UI, 47.9% nUI). By contrast, DT_XGB_5—trained with the most stringent single cutoff (5 µM)—was the most conservative, with only 30.7% UI (69.3% nUI). KNN_XGB_5–50 also skewed negative (40.3% UI, 59.7% nUI), consistent with its sensitivity to local neighborhoods in high-dimensional descriptor space, and RF_BORUTA_10–50 sat between these extremes (44.6% UI, 55.4% nUI). These patterns highlight complementary predictive behavior across algorithms, shaped by learning paradigm (tree-based vs. distance-based vs. boosting), feature selection strategy (Boruta, XGB, or none), and bioactivity cutoffs (single 5 µM vs. 5–50 µM cutoff).

To minimize model-specific bias and maximize the reliability of the ML-based filtering stage, a consensus voting strategy was adopted to integrate the predictions of the five classifiers. As shown in Figure 3, the individual models do not predict the UI/nUI classes in comparable proportions, reflecting intrinsic differences in sensitivity, specificity, and decision thresholds. For this reason, more permissive rules such as 3/5 or 4/5 votes tend to overweight the models that naturally overpredict one of the classes, introducing an implicit bias into the consensus and reducing the stability of the predictions across learning paradigms. The distribution of predicted UIs at different consensus levels illustrates this effect: 110,106 molecules were predicted as UI by at least one model, whereas 92,820, 93,526, and 105,602 compounds were simultaneously predicted by two, three, and four models, respectively. Importantly, adopting a 4/5 criterion produced over 20,000 additional molecules relative to the unanimous set, many of which displayed inconsistent or borderline classification patterns across models. In contrast, requiring agreement across all five classifiers ensured that only candidates whose predicted activity was stable across diverse algorithmic principles, were retained. Given the large scale of the screened library and the computational cost of the subsequent stages (ensemble docking, MD simulations, WT-MetaD), applying a strict unanimity rule at the MLVS level provided a clean and methodologically coherent subset for downstream refinement. Ultimately, 80,530 molecules were unanimously classified as UI by all five models and were thus selected for the next phase of the workflow, while all others were excluded from further consideration. It is important to note that this set is defined by pharmacophore compliance rather than strict chemical scaffold membership.

### 2.4. Ensemble-Docking Filtering

From the 80,530 molecules selected in the previous stage, a total of 26,342,100 docking poses were generated across 25 conformational frames of *Hp*U with Standard Precision (SP) method of Glide (Schrödinger Release 2023-3) [25]. For comparative purposes, three well-characterized UI were included as controls and processed using the same protocol: 2-{[1-(3,5-dimethylphenyl)-1H-imidazol-2-yl]sulfanyl}-N-hydroxyacetamide (DJM), β-mercaptoethanol (BME), and hydroxamic acid (AHA). These large-scale results were processed in R using an in-house library specifically developed to efficiently handle high-volume outputs from VS and ensemble docking experiments. This library enables the implementation of multiple data fusion strategies for ranking candidate compounds according to docking energy metrics.

Among the available data fusion approaches through the poses of the ligand, such as top-scoring, median, harmonic mean, arithmetic mean, and geometric mean (GM); the GM has been shown to provide the best correlation with experimental affinity values, as reported by Bajusz et al. [26]. Accordingly, the GM of docking energies across all poses was computed for each molecule, as well as for the three control inhibitors.

Figure S1 (Supplementary Information) depicts the distribution of docking energies across the 25 urease frames. The results show a consistent energy profile among frames, indicating that the conformational variability of *Hp*U does not drastically alter the relative energy distribution. The first frame exhibited the lowest median docking energies, suggesting that the initial conformation offers a slightly more permissive binding site; however, this does not necessarily reflect higher predictive accuracy, as the trend across frames remains coherent. The GM values of the controls are highlighted in the plot, maintaining the same order of inhibitory potency reported experimentally [27,28]. As summarized in Table S2, the GM docking energies were −6.75 kcal/mol for DJM, −5.09 kcal/mol for AHA, and −3.52 kcal/mol for BME. This ranking is consistent with their reported IC_50_ values and with the theoretical affinities estimated through the Cheng–Prusoff relationship previously applied by our group [14].

**Figure 4:**
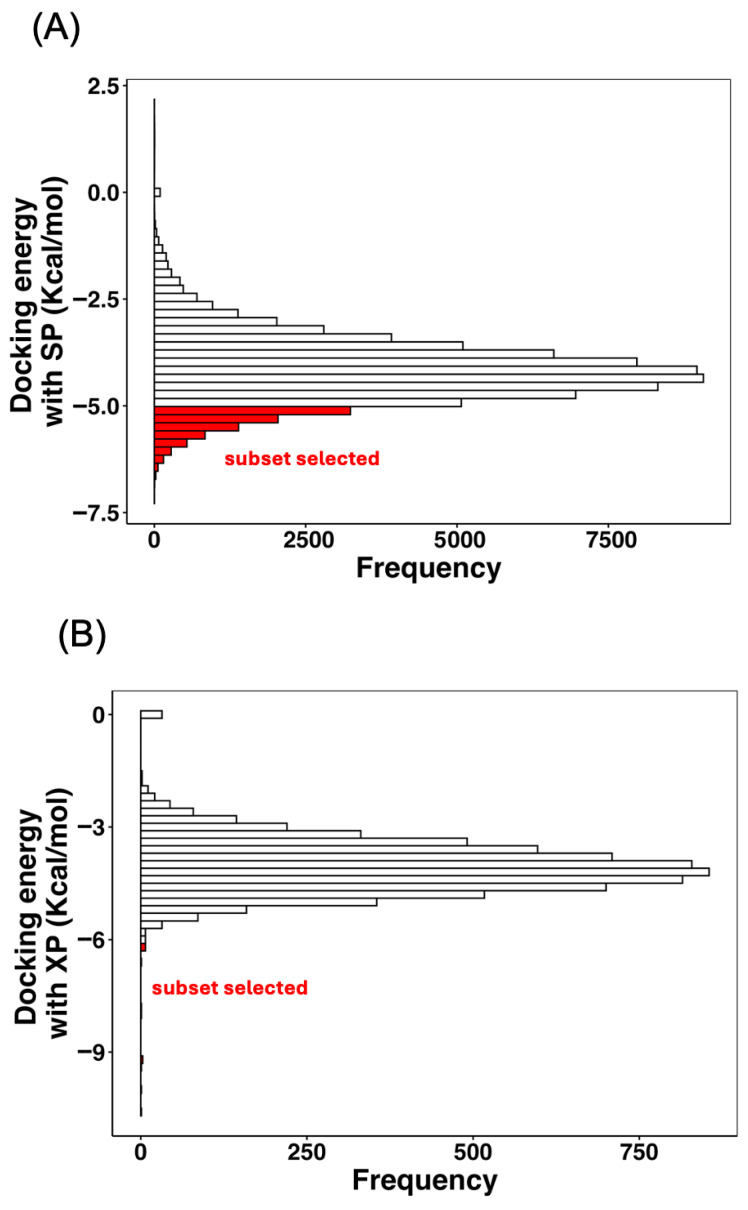
Histogram of GM docking energies with (**A**) SP and (**B**) XP modes for all screened compounds. The red-shaded region corresponds to the 7062 molecules with GM_docking values below the AHA control (−5.09 kcal/mol) in SP. Meanwhile, only 16 molecules in XP stage below the DJM control (−6.119 kcal/mol).

The GM for the ZINC subset (−4.15 kcal/mol) fell within the expected range for weak-to-moderate UI. To define an energy-based selection threshold, the GM value of each control was compared with that of all screened molecules. The number of ZINC compounds exhibiting a GM below each control was 66,112 for BME, 7062 for AHA, and only 7 for DJM. Given that DJM represents an excessively stringent reference and BME is too permissive, AHA was selected as the optimal cutoff criterion, providing a balance between selectivity and enrichment. Consequently, the 7062 molecules with GM docking energies lower than that of AHA were retained for the next stage. Figure 4A shows the histogram of GM docking energies for the entire ZINC subset, where the region below the AHA cutoff (−5.09 kcal/mol) is highlighted in red, representing the fraction of molecules selected for subsequent XP-docking refinement.

Figure S2 shows the distribution of Extra Precision (XP)-docking energies across the 25 conformational frames of *Hp*U. The overall pattern remained consistent with the SP-Glide results, yet the XP scoring function produced wider energy ranges and larger standard deviations (1.0–1.4 kcal/mol), consistent with its enhanced discriminatory capacity. Compared to SP, XP docking penalized less favorable orientations more strongly while improving separation among high-affinity candidates. Frames 0, 810, and 960 exhibited the lowest GM docking energies (−5.38, −4.98, and −4.65 kcal/mol, respectively), suggesting that these conformations correspond to more receptive catalytic pocket states. Nevertheless, the general trend across frames was coherent, indicating that conformational diversity did not introduce significant bias in energy distributions.

Figure 4B depicts the histogram of GM docking energies obtained from the XP stage. The distribution shows a sharp energetic concentration between −2 and −5 kcal/mol, with a narrow subset of high-affinity compounds reaching values below −6 kcal/mol. Statistical descriptors confirmed that XP results are more leptokurtic and right-skewed, indicating a sharper energetic differentiation within the ensemble. This behavior arises from the incorporation of explicit solvation and hydrophobic enclosure terms, which improve the physical realism of the scoring function. The overall energetic hierarchy of the control inhibitors remained unchanged: DJM > AHA > BME, but the gap between strong and moderate binders became more pronounced under XP refinement. As summarized in Table S3, the GM docking energies averaged −6.12 kcal/mol for DJM, −2.80 kcal/mol for AHA, and −2.71 kcal/mol for BME, while the ZINC database subset exhibited a mean of −4.06 kcal/mol. When comparing these distributions, 6861 molecules scored below BME, 6811 below AHA, and only 16 compounds achieved docking energies more favorable than DJM, which was therefore used as the final cutoff for candidate selection.

The 16 molecules were subjected to further evaluation. Visual inspection of their chemical structures revealed that eight compounds contained highly reactive or unstable functional groups, including nitro-heteroaromatic systems, which are typically associated with poor pharmacokinetic properties and potential chemical toxicity. These molecules were therefore discarded. Additionally, two molecules: ZINC000091409996 and ZINC000091409999 were identified as structurally identical, and one of them was removed to avoid redundancy. Consequently, a final set of seven candidate molecules (CA1–CA7) was retained for the next stage, as listed in Table S4, together with the three control inhibitors.

Ten ligands were subjected to QPLD (Schrödinger Release 2023-3) calculations across the same 25 conformational frames of *Hp*U used in the ensemble docking analyses. In this stage, QPLD was not applied as an additional filtering step but rather as a refinement and optimization procedure of the binding poses obtained in XP-Glide. This protocol incorporates quantum mechanically derived partial charges that account for electronic polarization effects within the binding pocket, providing a more accurate electrostatic description of protein–ligand interactions. The resulting optimized complexes, corresponding to the lowest-energy ensemble pose for each ligand, were subsequently selected as the starting structures for the MD simulations. This ensured that the dynamic analyses would begin from energetically and electronically consistent configurations, minimizing bias introduced by empirical docking charges.

### 2.5. Molecular Dynamics Stability of the Protein–Ligand Complexes

**Figure 5:**
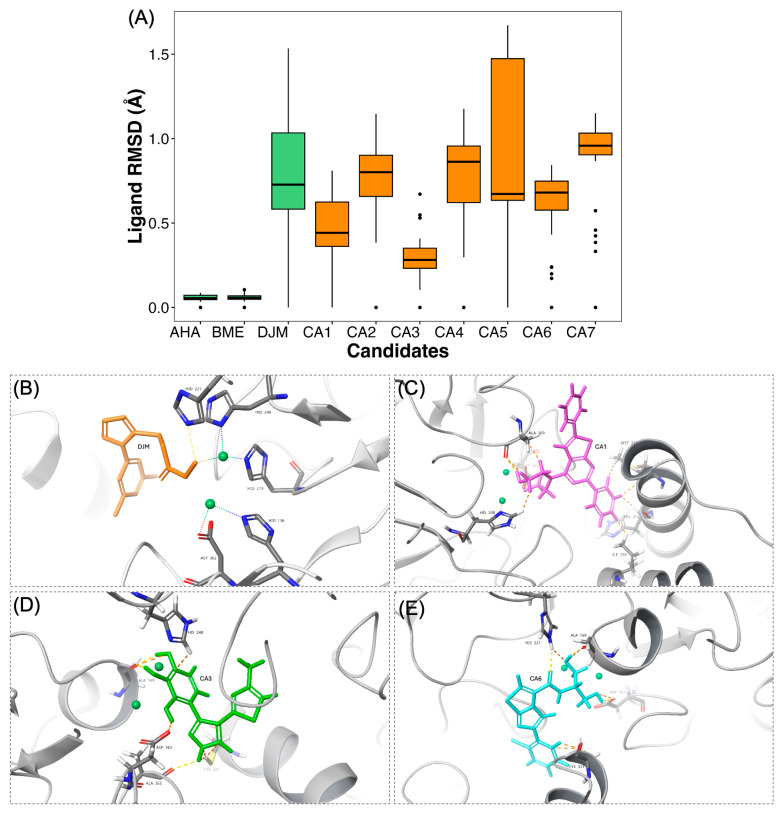
(**A**) Ligand RMSD values calculated over the final 20 ns of the MD simulations performed under unrestrained NPT conditions. Control inhibitors (AHA, BME, and DJM) and candidate molecules (CA1–CA7) are shown. Lower RMSD values indicate higher positional stability of the ligand within the catalytic pocket of *Hp*U. Panels (**B**–**E**) show the representative ligand conformations at the end of the unrestrained MD simulations for DJM (orange), CA1 (magenta), CA3 (green), and CA6 (cyan), respectively. Nickel ions are displayed as green spheres, and protein residues forming hydrogen bonds with the ligands are depicted in licorice representation, where oxygen atoms appear in red, nitrogen atoms in blue, and carbon atoms in gray, and sulfur atoms in yellow, following standard element-based coloring for structural visualization. These structural snapshots illustrate the degree of conformational retention or deviation relative to the initial bound pose and complement the dynamic stability analysis shown in panel (**A**).

To assess the structural stability of the urease–ligand complexes after QPLD optimization, all-atom MD simulations of 100 ns were performed under NPT conditions, applying progressively reduced positional restraints to the ligand. Only the final 20 ns of each trajectory, corresponding to the unrestrained segment, were analyzed to evaluate the intrinsic stability of the complexes under fully relaxed conditions. (Table S5, Figure 5).

The results show clear differences in the dynamic stability among the control inhibitors and the newly identified candidates. The control ligands AHA and BME exhibited minimal deviations (mean RMSD ≈ 0.057–0.058 Å, SD ≈ 0.018–0.019 Å), indicating that both compounds remained tightly coordinated within the catalytic pocket throughout the trajectory. This high rigidity is consistent with their small molecular size and their ability to maintain stable interactions with the two nickel ions (Ni^2+^) that define the urease active site. In contrast, the bulkier inhibitor DJM, despite being resolved within the catalytic site, displayed a significantly higher RMSD (mean ≈ 0.898 Å, SD ≈ 0.242 Å). This mobility likely reflects its larger conformational space and the flexible coordination of its hydroxamic acid moiety, which interacts alternately with the dinuclear Ni^2+^ cluster.

Among the candidate molecules (CA1–CA7), the observed RMSD values revealed distinct stability patterns. Compounds CA2, CA4, CA5 and CA7 showed the largest fluctuations (mean RMSD 0.76–0.95 Å, SD up to 0.47 Å), indicating greater intra-pocket mobility and loss of key stabilizing interactions, rather than full displacement from the binding cavity. The broader boxplot ranges observed for these ligands indicate multiple metastable orientations, possibly related to transient hydrogen-bond exchanges or steric clashes with the surrounding residues. Conversely, CA1, CA3, CA6, and CA7 maintained lower average deviations (0.29–0.62 Å) and narrower distributions, consistent with more persistent coordination to the dinuclear Ni^2+^ center and sustained interaction networks. Interestingly, CA3 displayed the lowest RMSD among all candidates (mean 0.29 Å), comparable to the stability of the controls, implying a well-defined binding mode stabilized by consistent interactions throughout the simulation. Such behavior could be indicative of a compact structure with favorable complementarity to the urease binding site. In contrast, the higher mobility of CA5, the largest of the candidates, may arise from the conformational flexibility of its peripheral substituents rather than from poor binding affinity per se. From a thermodynamic perspective, moderate ligand mobility is not inherently unfavorable, large and persistent fluctuations can disrupt metal coordination and compromise the effective residence time. CA2, CA4, CA5 and CA7 appear less stable under dynamic conditions, while CA1, CA3, and CA6, can be considered the most promising candidates based on this RMSD criterion.

The protein–ligand interaction profiles obtained from the final 20 ns of unrestrained MD simulations provided key insights into the molecular determinants responsible for the stability of the reference inhibitors and the top-ranked candidates. Figure S3 illustrates the dominant intermolecular contacts for AHA, BME, DJM, and the three most stable candidates (CA1, CA3, and CA6) identified from the RMSD analysis.

The control inhibitors AHA and BME maintained the canonical coordination pattern characteristic of UI. Both ligands remained tightly chelated to the dinuclear Ni^2+^ center, with AHA exhibiting a stable bidentate coordination through its hydroxamic oxygen atoms and BME maintaining a thiolate bridge between the two metal ions. In both cases, the nickel coordination was complemented by persistent hydrogen bonds with His136, His138, and His274, and by electrostatic interactions with Asp362. These interactions are consistent with the classical inhibitory mechanism in which the bound ligand neutralizes the positive charge density between the metal ions and impedes urea hydrolysis. The reference inhibitor DJM preserved a coordination geometry analogous to that reported experimentally [27], where the hydroxamic group bridges both nickel atoms, assisted by hydrogen bonding to Asp362 and Gly229. The persistence of these interactions throughout the trajectory explains the moderate RMSD values observed, confirming that DJM remains anchored despite minor conformational breathing of its aromatic scaffold.

Among the new candidates, the three selected ligands—CA1, CA3, and CA6—displayed consistent and complementary binding modes that recapitulate several features of the control inhibitors while introducing new stabilizing interactions.

- CA1 established dual coordination with the Ni^2+^ ions via its triazole nitrogen atoms and a nearby hydroxyl group, while forming stable hydrogen bonds with Asp362 and Ala169, and hydrophobic contacts with adjacent residues. This coordination pattern mimics the binding mode of AHA but extends further into the hydrophobic subpocket, providing additional van der Waals stabilization.
- CA3 exhibited the most stable binding pose, consistent with its lowest RMSD values. The compound engaged one Ni^2+^ ions through its heteroaromatic ring and thiol moiety, maintaining long-lived hydrogen bonds with His221, Asp362, and Ala165. The presence of dual hydroxyl substituents allowed the ligand to anchor simultaneously to polar and hydrophobic regions, generating a well-balanced interaction network that likely underpins its superior thermodynamic stability.
- CA6 also adopted a chelating orientation toward the nickel ion, stabilized by hydrogen bonds with Asp362 and Cys321 and cation-π with His322. Despite a more extended structure, CA6 retained an orientation like that of DJM, preserving its interactions during the unrestrained stage and suggesting that it may act as a competitive inhibitor capable of occupying the same catalytic niche.

Collectively, the final MD snapshots in Figure 5C–E confirm that CA1, CA3, and CA6 exhibit strong geometrical complementarity to the *Hp*U catalytic pocket, reproduce the essential Ni^2+^ coordination observed for potent inhibitors (Figure 5B), and preserve favorable interactions during unrestrained dynamics. These structural observations, together with their low RMSD values, strongly support the selection of CA1, CA3, and CA6 as the most robust urease-binding candidates for subsequent free-energy and WT-MetaD analyses.

### 2.6. Well-Tempered Metadynamics Analysis

The distributions of the collective variables (CVs) sampled during the WT-MetaD simulations provide a first qualitative description of the conformational space explored by the urease–ligand complexes. Figure S4 summarizes the histograms for the control inhibitors, while Figure S5 shows the corresponding distributions for the selected candidates CA1, CA3, and CA6. In these analyses, CV1 (pink) is the distance between the ligand geometric center of mass (COM) and the catalytic Ni^2+^ ions; CV2 (blue) is the distance between the COM of key active-site residues (KCX219, H274, C321, D362, A365; heavy atoms only) and the COM of ligand heavy atoms.

For the control inhibitors (Figure S4), the histograms reveal distinct mobility patterns consistent with their experimental behavior.

- AHA (A–C): Narrow CV1 at ~1.5–3.0 Å and short CV2 with tight spread indicate a compact pose that remains close to both the metal center and the residue cluster.
- DJM (D–F): Broader CV1 (~4.0–6.5 Å) and CV2 shifts to longer distances episodically, consistent with partial breathing/tilting of the hydroxamate arm that moves the ligand away from the residue cluster while remaining near the Ni^2+^ core.
- BME (G–I): Exhibited narrower CV1 distributions centered at shorter distances (~2.0–3.0 Å) and broader CV2 extending up to ~5 Å. This pattern suggests that BME remains close to the Ni^2+^ ions but explores larger fluctuations relative to the surrounding residue cluster. Such behavior is consistent with a shallow but persistent anchoring mode, where the ligand oscillates near the catalytic metals without achieving deep burial into the active-site network. This partially uncoordinated dynamic agrees with its weak inhibitory potency (IC_50_ ≈ 13,500 μM) and indicates a transient, low-residence binding profile.

In contrast, the candidate molecules (Figure S5) exhibited broader and more diverse sampling of the CV space, reflecting their higher molecular complexity and potential to explore multiple metastable binding modes.

- CA1 (A–C): CV1 moderate with CV2 spanning mid–long distances, suggesting alternation between a buried state and a mouth/entrance-proximal pose rebinding/transient excursions along the pocket rim.
- CA3 (D–F): Well-defined CV1 around ~5 Å with CV2 clustered at intermediate distances; across replicas both CVs show narrow peaks, indicating a single dominant basin where the ligand stays close to the catalytic residues while maintaining a stable depth—consistent with its low RMSD and persistent contacts.
- CA6 (G–I): Narrow CV1 with CV2 toggling between intermediate and longer values, pointing to transitions between a compact, residue-engaged state and a partially disengaged configuration near the pocket entrance; this matches its intermediate RMSD/stability profile.

Overall, the CV histograms across all replicas confirm that the selected reaction coordinates effectively capture the essential motions associated with ligand retention and release in the urease catalytic pocket. The distinct separation of CV1–CV2 peaks between high- and low-affinity inhibitors underscores the ability of these variables to discriminate between compact, metal-centered coordination and more peripheral, labile interactions. When comparing DJM (IC_50_ = 19.6 μM) and BME (IC_50_ = 13,500 μM), their WT-MetaD profiles reveal that BME predominantly samples short CV1 distances (~1–3 Å), consistent with a shallow, metal-adjacent pose, while DJM exhibits a clear shift toward longer CV1 values (~4–6.5 Å), reflecting deeper accommodation within the catalytic pocket and enhanced engagement with surrounding residues. Similarly, the candidate ligands CA6, CA3, and, to a lesser extent, CA1 reproduce the DJM-like CV1 distribution across replicas, indicating a comparable degree of burial and stability within the active site. Although CV2 shows partially overlapping ranges among all systems, with BME rarely exceeding ~4 Å and DJM or the candidates occasionally reaching beyond 7 Å, the predominant CV1 patterns of CA6 and CA3 align more closely with the potent inhibitor DJM than with the weak, surface-biased BME. These results suggest that CA6 and CA3, and to a lesser degree CA1, adopt binding modes characterized by stable coordination to the Ni^2+^ cluster and consistent interaction with the active-site residues, supporting their prioritization for the subsequent free-energy surface reconstruction.

The replicate-averaged time series (Figure S6A) reveal two distinct dynamical regimes consistent with the experimental inhibitory hierarchy. The weak inhibitors AHA and BME maintained short and steady CV1 distances (~2–3 Å), consistent with a shallow, metal-proximal binding mode. Conversely, DJM and the top-ranked candidates (CA1, CA3, CA6) showed larger, well-stabilized CV1 values (~5–6.5 Å), reflecting persistent burial within the catalytic pocket. For CV2 (Figure S6B), which tracks the distance between the ligand and the cluster of catalytic residues (KCX219, H274, C321, D362, A365). The weak inhibitors again occupied shorter and more fluctuating ranges (~2–3 Å), whereas DJM and the candidates sampled broader distributions centered at 4–6 Å, with limited drift over time. Together, these trajectories demonstrate that our candidates reproduce the dynamic signature of the potent inhibitor DJM, stable, buried coordination and minimal drift, supporting their selection as the most promising ligands.

**Figure 6:**
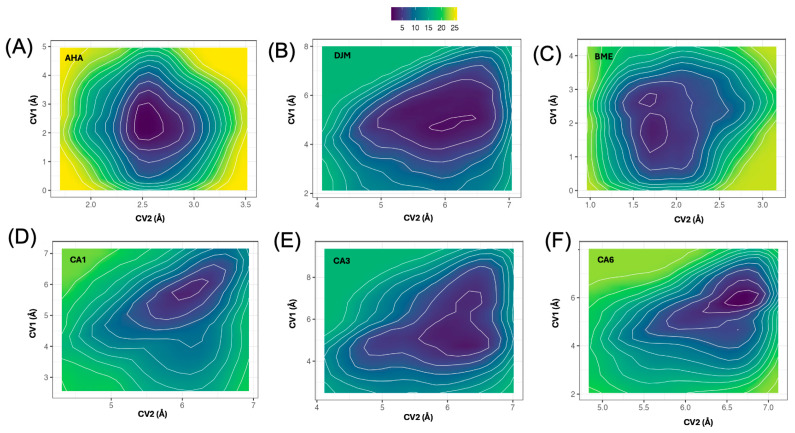
Two-dimensional free-energy surfaces (FES) reconstructed from the WT-MetaD simulations of the control inhibitors (AHA, BME, DJM) and the selected candidates (CA1, CA3, CA6). The FES are plotted as functions of CV1 (distance between the center of mass (COM) of ligand heavy atoms and the Ni^2+^ cluster) and CV2 (distance between the COM of the ligand and the COM of the key catalytic residues KCX219, H274, C321, D362, and A365). Darker regions denote lower free energy. (**A**) FES for AHA. (**B**) FES for DJM. (**C**) FES for BME. (**D**) FES for candidate CA1. (**E**) FES for candidate CA3. (**F**) FES for candidate CA6.

The 2D free-energy surface (FES) mapped over CV1 and CV2 (Figure 6) reveals two clearly separated binding regimes. The weak controls (AHA, BME) show compact basins located at low CV1 (≈1.6–2.8 Å) and low–mid CV2 (≈1–3 Å), consistent with shallow, metal-proximal poses that remain close to the Ni^2+^ center and the nearest catalytic residues. By contrast, DJM and the candidates populate deeper basins shifted to higher CV1 (≈5.5–6.8 Å) and mid–high CV2 (≈4.5–6.5 Å), reflecting burial within the pocket and sustained engagement with the residue cluster (KCX219, H274, C321, D362). Among the candidates, CA6 presents the deepest and most localized minimum (CV1 ≈ 6.7 Å, CV2 ≈ 6 Å), forming a tight basin comparable to—if not slightly more stabilized than—DJM (minimum near CV1 ≈ 6.2 Å, CV2 ≈ 5.2 Å). CA3 shows a pronounced minimum around CV1 ≈ 6.0 Å/CV2 ≈ 4.8–5.0 Å with an elongated valley toward larger CV2, indicating a coupled motion where residue engagement modulates depth without compromising pocket burial. CA1 also centers its minimum at CV1 ≈ 6.2–6.6 Å, but the basin is shallower and tilted toward longer CV2, suggesting easier excursions toward the pocket mouth. The 1D projections support these trends. Along CV1 (Figure S7A), the well depths order roughly as CA3 ≈ DJM ≥ CA6 > CA1 ≫ AHA > BME, placing CA3, CA6 as the most stabilized deep-pocket states. Along CV2 (Figure S7B), the minima occur near ~5–5.5 Å for DJM/CA6/CA3, while CA1 sits slightly lower (~4.5–5 Å) and AHA/BME at ~2–2.5 Å, consistent with their more superficial engagement. In qualitative WT-MetaD terms, deeper, narrower wells and higher barriers along the escape direction (increasing CV1 and/or CV2) correlate with greater kinetic persistence (longer residence time) and stronger apparent affinity, as discussed in metadynamics studies of protein–ligand dissociation [29,30,31]. Overall, CA6 and CA3 reproduce the DJM-like deep, residue-engaged basin, whereas CA1 shows intermediate stabilization and a more accessible path toward partial disengagement. These landscapes justify prioritizing CA6 and CA3 for future experimental follow-up, with CA1 retained as a promising, but less kinetically robust backup.

### 2.7. Chemical Space Analysis

**Figure 7:**
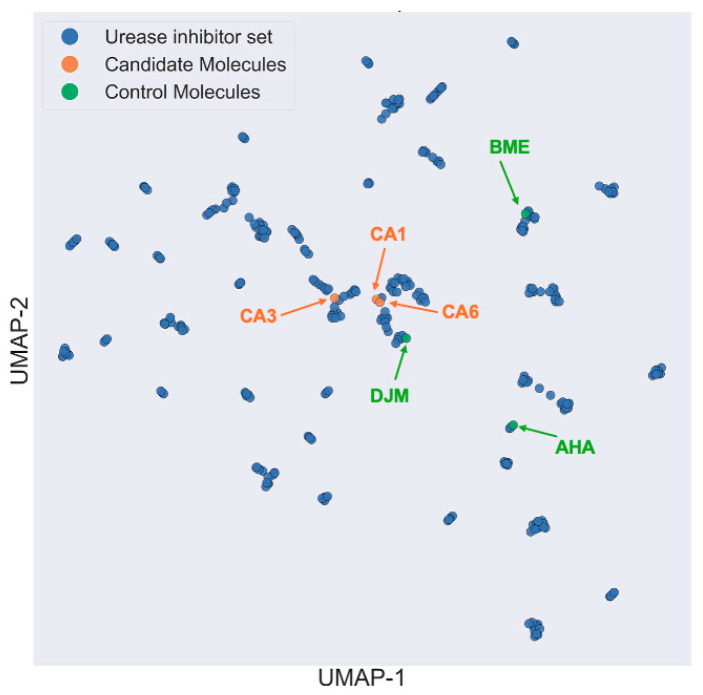
Two-dimensional UMAP of the chemical space comprising reported *Hp*U inhibitors (blue), control molecules (green), and the three top-ranked candidate ligands (orange). The map was generated using the ChemPlot Python library based on molecular fingerprint descriptors.

Figure 7 shows the two-dimensional map of the chemical space generated using the UMAP algorithm implemented in the ChemPlot Python library. The dataset includes reported *Hp*U inhibitors from public databases (ChEMBL, PubChem, and BindingDB), the three control molecules (AHA, BME, and DJM), and the three top-ranked candidate ligands (CA1, CA3, and CA6). The visualization reveals that the candidate molecules cluster in proximity to DJM, a potent UI, suggesting that their overall molecular architecture and substructural patterns share higher similarity with potent inhibitors than with weak binders such as BME. This observation is consistent with previous energetic analyses, but is conducted in terms of topological and descriptor-based similarity rather than binding energy. The Uniform Manifold Approximation and Projection (UMAP) projection captures nonlinear relationships among molecular fingerprints by preserving local neighborhood distances within a reduced dimensional space, making it particularly effective for visualizing structural analogies across diverse chemical scaffolds. As reported by Sorkun et al. [32], this approach offers advantages over linear methods such as Principal Component Analysis (PCA) by maintaining the continuity of local clusters and reducing information loss in high-dimensional chemical datasets. In this context, the positioning of CA1 and CA6 near DJM supports their classification as chemically and functionally close analogs within the UI landscape, whereas CA3 occupies an intermediate region, bridging the space between DJM-like structures.

To further contextualize these findings, we performed a nearest-neighbor analysis within the UMAP space to quantify the structural relationships between each candidate ligand and experimentally reported *Hp*U inhibitor (Table S7). For CA1, CA3, and CA6, the five closest neighbors in descriptor space were exclusively hydroxamate-based ligands, including DJM-like scaffolds, reflecting the absence of potent non-hydroxamic analogs in the vicinity of the candidates within the chemical landscape. These neighbors exhibit experimentally reported IC_50_ values spanning broad potency ranges, and their mean IC_50_ values (CA1: 2469.46 µM; CA6: 34.28 µM; CA3: 152.45 µM) were computed solely as descriptive indicators of the historical activity associated with each local chemical environment. Importantly, these averages are not intended as predictive estimates of the candidates’ potency, but rather as contextual descriptors showing that the structural neighborhoods surrounding CA6 and CA3 are associated with comparatively more potent hydroxamate inhibitors than those near CA1. Collectively, the absence of strong non-hydroxamic neighbors and the proximity of the candidates to well-established hydroxamate chemotypes further support the rationale for using DJM as the potency benchmark in this study and highlight that the triazole-based candidates cluster most closely with the chemical scaffolds historically linked to high urease inhibitory activity.

Taken together, the chemical space mapping provides a complementary, global validation of our computational pipeline: compounds that emerged as top energetic and dynamical performers also occupy chemically coherent regions associated with potent inhibitors. This convergence between biophysical and cheminformatics descriptors reinforces the robustness of the multistage strategy—spanning pharmacophore modeling, docking, enhanced sampling, and data-driven chemical analysis—as a rational framework for identifying novel UI. Future work will include in vitro evaluation of CA1, CA3, and CA6 to confirm their inhibitory activity and refine structure–activity relationships for further lead optimization.

## 3. Materials and Methods

### 3.1. Curation of the ZINC15 Database

The initial compound library was obtained from the ZINC15 database [33], a widely recognized repository of commercially available small molecules. An initial set of 7,153,060 compounds was curated by applying preliminary physicochemical filters, retaining only those with a MW of ≤500 g/mol and a LogP of ≤5. To eliminate redundancy, duplicates and tautomers were removed. All compounds were processed using LigPrep (Schrödinger Release 2023-3) [34] to generate up to two stereochemically plausible 3D conformations per compound.

Subsequent pharmacokinetic filtering followed the same ADMET-based workflow previously reported in Valenzuela-Hormazábal et al. (2024) [14]. Briefly, QikProp descriptors (Schrödinger Release 2023-3) were used to apply:

(i) Lipinski’s Rule of Five [35], allowing a maximum of one violation;
(ii) Jorgensen’s Rule of Three [36], requiring QPlogS > −5.7, QPPCaco > 22 nm/s, and ≤7 likely primary metabolites; and
(iii) Veber’s Rule [37], retaining compounds with ≤10 rotatable bonds and PSA ≤ 140 Å^2^.

### 3.2. Generation of Pharmacophore Hypotheses

A PBVS approach was employed to enrich the database with compounds exhibiting potential ureolytic activity. A comprehensive literature review was conducted via Web of Science (WoS) using the keywords “urease inhibitors”, “*Helicobacter pylori*” and “triazoles,” focusing on articles published post-2010. This search yielded 147 triazole-based urease inhibitors, which were classified by activity: 97 active (IC_50_ ≤ 25 µM) and 39 inactive molecules (IC_50_ > 25 µM) [38,39,40,41,42,43,44,45,46,47].

Pharmacophore models were generated using the Phase module (Schrödinger Release 2023-3) [48]. Ten pharmacophore hypotheses were generated, capturing essential features such as HB acceptors (A) and HB donors (D), hydrophobic groups (H), aromatic rings (R), negative (N) and positive (P) ionic. The optimal hypothesis, AHRR1, was selected based on the BEDROC metric, achieving a score of 0.6222. AHRR1 featured four pharmacophoric points with maximal separation between active and inactive molecules, enhancing enrichment capability.

Application of the best model to the curated dataset of ZINC molecules yielded 2,485,924 hits. Further refinement was performed using the PSS. Compounds scoring above the third quartile (Q3 ≥ 0.797) were retained for subsequent analysis. The pharmacophore filter selects molecules matching the triazole-derived interaction hypothesis, not exclusively triazole scaffolds.

### 3.3. Machine Learning Filter

In a previous study by our group [20], 252 distinct ML models were trained to classify molecules as UI or nUI based on physicochemical descriptors. These models were constructed by systematically combining seven ML algorithms (DT, random forest (RF), XGBoost (XGB), k-nearest neighbor (kNN), logistic regression (LR), support vector machine (SVM), and naïve Bayes (NB)), three feature selection strategies (Boruta, XGB, and no feature selection), and six bioactivity categorization schemes based on different IC_50_ thresholds, including both single- and dual-cutoff strategies to define gray zones. Performance evaluation relied on repeated cross-validation and was benchmarked using the Matthews correlation coefficient (MCC) and area under the receiver operating characteristic curve (AUC-ROC). Among these, tree-based methods consistently achieved the highest predictive performance, with AUC values exceeding 0.93 as reported in [20].

For the present study, the five best-performing models reported previously, shown in Table 1: DT_BORUTA_5–50, XGB_nFS_25–50, DT_XGB_5, KNN_XGB_5–50, and RF_BORUTA_10–50 were selected for compound filtering. In this nomenclature, the first component indicates the classification algorithm, the second specifies the attribute selection method, and the third denotes the bioactivity cutoff applied to distinguish UI from nUI in the training dataset.

To prepare the dataset, the compounds retained after pharmacophore screening were converted to SDF format, and their physicochemical descriptors were calculated using the rCDK package (R environment, v. 3.6.0) [49]. This process generated a matrix of 623,350 molecules × 287 descriptors, which served as input for ML-based prediction. Each model was independently applied to classify compounds as potential UI or nUI. Only those molecules consistently predicted as UI by all five models were retained for subsequent stages of the pipeline, ensuring a high-confidence subset enriched in triazole-derived UI candidates.

### 3.4. Ensemble-Docking with SP Glide

Currently, four resolved structures of *Hp*U are available in the Protein Data Bank (PDB IDs: 6ZJA, 6QSU, 1E9Y, and 1E9Z) [27,50]. With the exception of 1E9Z, the remaining three were determined with known inhibitors: DJM, BME, and AHA, bound at the catalytic site. Among them, 6ZJA exhibits the highest resolution (2.0 Å) and was selected as the reference structure for this stage. To explore the conformational variability of the active site, all-atom MD simulations were performed on 6ZJA over a total timescale of 2 μs.

Prior to simulation, the dodecameric enzyme was reduced to a representative catalytic unit (monomer B) and one accessory unit (monomer A). The protein structure was prepared at the optimal pH for urease activity using the Protein Preparation Wizard (Schrödinger Release 2023-3), adding hydrogen atoms to stabilize the protein architecture. The electron microscopy references of the ligand DJM and the two Ni^2+^ ions were retained during preparation. Partial charges and bond orders were assigned using the OPLS3e force field [51]. The system was energy-minimized, restricting minimization to hydrogen atoms, and subsequently solvated in an SPC water box with Na^+^ and Cl^−^ ions for charge neutralization. Default equilibration protocols in Desmond were applied prior to production runs, which were carried out under NPT ensemble conditions at 300 K and 1 atm with periodic boundary conditions.

Trajectory files (*.trj) were converted to *.dcd format for downstream analysis with the R package Bio3D [52]. The root mean square deviation (RMSD) of residues within the catalytic site was calculated for all frames of the 2 μs trajectory, and an RMSD-based hierarchical clustering was performed to identify conformational microstates sampled by the enzyme. The optimal range of cluster numbers was assessed using both elbow analysis of within-cluster sum of squares (WSS) and average silhouette width (Supplementary Figure S9A,B). Both diagnostics indicated a broad stability plateau for *k* ≥ 10, suggesting that any number of clusters within this region provides a reasonable description of the conformational ensemble without overfragmentation. Based on this plateau, 25 clusters were selected as a balanced representation of catalytic-site diversity. A representative centroid frame from each cluster was extracted (Supplementary Figure S9C), yielding 25 conformational states of *Hp*U that were subsequently used for ensemble docking.

The compounds retained from the previous stage were prepared with LigPrep at pH 7.0, generating up to two tautomers and two protonation states per molecule. Ensemble docking was then performed by generating 25 receptor grids centered on the Ni^2+^ ions in the catalytic site, one for each representative conformer. Docking was carried out with Glide in SP mode under the OPLS3e force field, generating up to 10 poses per molecule per receptor conformation. This yielded approximately 5.4 million docking poses in total. The same procedure was applied to the control inhibitors DJM, AHA, and BME. The docking output data were subsequently processed in R using an in-house library specifically developed to efficiently handle large-scale VS and ensemble docking datasets. This library enables the calculation of multiple statistical metrics to prioritize candidate molecules based on docking energies.

In particular, the GM docking score across all poses was computed for each compound as well as for the control inhibitors. To establish a rational cutoff for candidate selection, the GM scores of the three controls were compared. Only a very small fraction of compounds outperformed DJM, whereas a larger proportion surpassed AHA, and an even greater number exceeded BME. Consequently, the value obtained for AHA was selected as the cutoff threshold, retaining only those molecules with superior GM docking scores for progression to the next stage.

### 3.5. Ensemble-Docking with XP Glide

Following SP-docking, the molecules retained based on their GM docking scores below the AHA control were subjected to a more rigorous XP [53] docking analysis of Glide (Schrödinger Release 2023-3). The XP protocol was applied to the same 25 conformational frames of *Hp*U generated from MD clustering. For each compound, up to 10 poses were generated per frame, and the GM of XP docking scores was calculated to provide a robust affinity estimate across conformational states.

This refinement step aimed to improve candidate prioritization by selecting those compounds with binding affinities stronger than the DJM control molecule, which represents one of the most potent UI reported. In this way, only candidates consistently outperforming DJM across multiple conformers were advanced to the subsequent stage of analysis.

### 3.6. Quantum Polarized Ligand Docking

Compounds exhibiting GM docking scores superior to the DJM control were subjected to visual inspection in Maestro-Schrödinger Suite to exclude structures with undesirable chemical features. Molecules displaying high chemical reactivity, unstable functional groups, or moieties associated with potential toxicity (e.g., nitro-heteroaromatic systems with multiple electron-withdrawing substituents) were discarded at this stage.

The remaining molecules were further refined using the QPLD protocol (Schrödinger Release 2023-3), which provides a more accurate representation of protein–ligand electrostatics compared to conventional docking. Unlike classical docking approaches that rely on fixed empirical charges, QPLD incorporates quantum mechanically derived partial charges for ligands, thereby accounting for polarization effects induced by the receptor environment. This resulted in a more realistic estimation of binding affinity and improved discrimination of true binders from false positives. To enhance robustness, an ensemble-QPLD strategy was employed, in which all 25 conformational frames of *Hp*U generated in the previous stages were used as receptor models.

In the present workflow, QPLD was not used as an additional filtering or ranking step, but rather as a refinement stage applied only to the shortlisted complexes that had already passed pharmacophore-based, ML-based screening and undergone ensemble docking. The objective of QPLD was to optimize ligand partial charges under the electrostatic influence of the dinuclear Ni^2+^ center and to improve the accuracy of metal coordination prior to MD simulations, thereby enhancing the physicochemical fidelity of the selected complexes rather than altering their prioritization.

Ligand partial charges were computed using the Jaguar quantum chemistry package (Schrödinger Release 2023-3) [54] with the B3LYP functional [55] and the lacvp* basis set, without application of an implicit solvation model. These quantum-derived charges were then mapped onto the ligands during the PULL stage of the QPLD workflow. Redocking was subsequently carried out in Glide XP mode, employing an RMSD cutoff of 0.5 Å, a maximum atomic displacement of 1.3 Å, and a ligand van der Waals (LVDW) scaling factor of 0.8. Up to 77 poses were initially generated per ligand, which were reduced to a maximum of 10 poses based on Glide scoring criteria. Importantly, the QM-derived charges were retained during redocking to ensure explicit consideration of electrostatic polarization throughout the docking process. The same procedure was applied to the control inhibitors DJM, AHA, and BME. For each molecule, including the controls, all ensemble-generated protein–ligand complexes were compared, and the lowest-energy pose was selected to be carried forward into the next stage of the protocol.

### 3.7. MD Simulations of Protein–Ligand Complexes

The lowest-energy ensemble poses identified from the QPLD stage were subjected to all-atom MD simulations to evaluate the stability and dynamic behavior of the protein–ligand complexes under near-physiological conditions. Each system was simulated in the NPT ensemble (T = 300 K, *p* = 1 atm) for a total of 100 ns.

To gradually relax the complexes and prevent artificial distortions of the ligand binding mode, a staged restraint protocol was applied. In the first production run (20 ns), a positional restraint of 10 kcal/(mol·Å^2^) was imposed on the ligand heavy atoms. This restraint was progressively reduced in subsequent stages: 5 kcal/(mol·Å^2^) during the second 30 ns run, 2 kcal/(mol·Å^2^) during the third 30 ns run and completely removed in the final 20 ns run. This stepwise relaxation strategy allows the system to retain key protein–ligand contacts initially observed in docking, while progressively permitting conformational adaptation of both ligand and binding site residues. In this way, potentially artifactual displacements caused by abrupt ligand release are avoided, favoring the stabilization of interactions that genuinely dominate the complex under dynamic equilibrium.

Importantly, the two Ni^2+^ ions present in the catalytic site were explicitly included in all simulations. Their coordination with the surrounding protein residues was maintained according to the electron microscopy reference structure, thereby preserving the integrity of the metalloenzyme active site throughout the trajectories. All other simulation parameters (force field, solvation model, ion neutralization, and equilibration protocol) were identical to those described in Section 2.4 (Ensemble Docking with SP-Glide).

Upon completion of the simulations, conformational dynamics parameters (RMSD, RMSF) and intermolecular interactions (hydrogen bonds, hydrophobic contacts, and metal coordination) were analyzed to compare the behavior of the candidate complexes relative to the three control inhibitors. Finally, the last frame of each simulated complex was extracted and selected as the representative structure for the subsequent stage of the protocol.

### 3.8. Well-Tempered MetaDynamics Simulations of Protein–Ligand Complexes

To investigate the unbinding mechanism of candidate inhibitors and to enable a quantitative comparison of their relative stability within the catalytic site of *Hp*U, we performed WT-MetaD simulations. All simulations (candidates and controls) were carried out in the NVT ensemble at 300 K for a total of 100 ns in triplicate. Gaussian bias hills of 0.06 kcal/mol were deposited at 1 ps intervals along the predefined CVs, with widths of 0.10 Å. The well-tempering parameter was set to kTemp = 8.4 kcal/mol, corresponding to a bias factor of approximately γ ≈ 15.2 at 300 K.

Two CVs were selected to describe the essential degrees of freedom governing ligand exit:

1. CV1 (metal–ligand coordination distance): distance between the center of mass (COM) of the two nickel ions and the COM of the ligand heavy atoms. An upper wall of 8 Å was applied to limit unphysical excursions.
2. CV2 (pocket–ligand distance): distance between the COM of key active-site residues (KCX219, H274, C321, D362, and A365, heavy atoms only) and the COM of the ligand heavy atoms. An upper wall of 15 Å was enforced to ensure adequate sampling of the exit pathway while avoiding instability.

These CVs were selected to capture the two dominant processes in the unbinding mechanism: loss of metal coordination (CV1) and radial displacement of the ligand from the catalytic pocket (CV2).

During each simulation, the instantaneous values of the CVs and bias potential were recorded in .*cvseq* and .*kerseq* files, respectively. Time-series plots of CV1 and CV2 were generated to monitor sampling extent and stability, while histograms of each CV were computed to quantify configurational coverage. These diagnostics were produced for every replica and are reported in the Supplementary Information (Figures S4–S7). The bias potential accumulated along the trajectories was used to reconstruct the time-dependent FES. For a system defined by the collective variables s = (s_1_, s_2_), the free energy is expressed as:

$$F\left(s_{1},s_{2}\right)\propto -\sum_{i}h_{i}exp\left(-\frac{{\left(s_{1}-c_{1i}\right)}^{2}+{\left(s_{2}-c_{2i}\right)}^{2}}{2\sigma^{2}}\right)$$

where $h_{i}$ and $c_{i}$ are the height and center of the i-th Gaussian, σ is the hill width and ***CV*1** = $s_{1}$ and ***CV*2** ${=s}_{1}$. Block reconstructions were performed at 25, 50, 75, and 100 ns to evaluate convergence. Stabilization of the FES across consecutive blocks was taken as evidence of reliable sampling.

After convergence validation, the final FES from each replica was aligned to a common 2D grid and offset to its putative global minimum (Δ*F* = 0 kcal·mol^−1^). Replica surfaces were resampled on the intersection of their CV ranges and combined to yield the mean free-energy surface (*F_mean_*) and its standard error (*F_SEM_*):

$$F_{mean}\left(s_{1},s_{2}\right)=\frac{1}{N}\sum_{r=1}^{N}F_{r}\left(s_{1},s_{2}\right)$$

$$F_{SEM}\left(s_{1},s_{2}\right)=\frac{\sigma \left[F_{r}\left(s_{1},s_{2}\right)\right]}{\sqrt{N}}$$

with *N* = 3 replicas. The resulting 2D surfaces were visualized as heat maps with contour lines representing iso-energetic levels of Δ*F*. To obtain one-dimensional free-energy profiles for each CV, Boltzmann marginalization was applied to the replica-averaged surface:

$$F_{proj}(x)=-k_{B}T\ln{\langle e^{-F(x,y)/k_{B}T}\rangle }_{y}$$

where *k_B_T* is the thermal energy at 300 K. The 1D profiles along CV1 and CV2 characterize the energetic barriers associated with metal–ligand dissociation and ligand displacement from the pocket, respectively.

The final set of analyses thus comprised:

(i) time-series of CV1 and CV2 (per replica),

(ii) histograms of CV1 and CV2 (per replica),

(iii) 1D free-energy profiles of CV1 and CV2 (replica-averaged), and

(iv) the 2D free-energy surface (mean ± SEM).

Together, these plots describe the collective motion and free-energy barriers governing ligand unbinding and enable quantitative comparison among candidate inhibitors.

### 3.9. Chemical Space Comparison of Reported Urease Inhibitors and Candidate Molecules

To contextualize our results, the chemical space of the candidate compounds was compared with that of reported *Hp*U inhibitors. A systematic search was performed in the three major compound databases: ChEMBL [56], PubChem [57], and BindingDB [58] using the keywords “urease inhibitor” AND “*Helicobacter pylori*”. From the retrieved records, SMILES strings were extracted and canonicalized. Duplicates were removed, and inhibitors lacking experimental activity data (IC_50_ or K_i_) were discarded, following a workflow similar to the exploratory data analysis (EDA) previously reported by our group [14].

The curated dataset of reported inhibitors was then combined with our candidate molecules for comparative analysis. Using the ChemPlot Python library (https://chemplot.readthedocs.io/en/latest/ (accessed on 3 October 2025)) [32]. The UMAP algorithm was used for dimensionality reduction and visualization, allowing the relative positioning of our candidates to be assessed within the broader chemical landscape of known UI–like compounds.

### 3.10. ADMT Predictions

Predicted absorption, distribution, metabolism, and toxicity parameters for the final three candidate molecules (CA1, CA3, and CA6) were computed using ADMETlab 3.0 (https://dmetlab3.scbdd.com/ (accessed on 20 November 2025)) [59]. The analysis included physicochemical descriptors (MW, TPSA, logP, logS, rotatable bonds), absorption properties (Caco-2, MDCK, HIA, P-gp interactions), distribution metrics (BBB penetration probability, PPB, VDss), metabolism-related predictions for major CYP isoforms, and key toxicity endpoints (hERG blockade, DILI, hepatotoxicity, Ames mutagenicity, and genotoxicity). A curated subset of these descriptors judged most relevant for early-stage in vitro evaluation is reported in Table S8.

## 4. Conclusions

This study implemented a multistage VS pipeline, integrating pharmacophore modeling, ML classification, ensemble docking, and atomistic simulation techniques to identify novel triazole-based inhibitors targeting *Hp*U. The protocol successfully combined ligand-based and structure-based strategies, progressively refining a large chemical library from over seven million compounds to a final subset of three high-confidence candidates. The pharmacophore-based filtering effectively captured key interaction motifs associated with triazole-derived UI, while the ML stage, based on five previously validated classification models, ensured the consistent selection of molecules with physicochemical patterns predictive of ureolytic inhibition. Ensemble docking across 25 conformational frames of *Hp*U, followed by energy-based data fusion using geometric mean scoring, allowed a statistically robust ranking of candidate ligands. This approach reproduced the experimental potency hierarchy of known inhibitors, DJM, AHA, and BME, thereby validating the computational framework. Subsequent refinement by QPLD confirmed the stability of metal coordination and key hydrogen-bonding interactions within the catalytic site. MD simulations under progressively relaxed restraints demonstrated that the shortlisted complexes maintained stable conformations comparable to the most potent control (DJM). Ligand RMSD analyses identified CA1, CA3, and CA6 as the most stable complexes, exhibiting persistent coordination with the nickel ions and catalytic residues. Finally, WT-MetaD simulations provided a detailed description of the free-energy landscape governing ligand binding and unbinding. The reconstructed 2D free-energy surfaces and 1D projections revealed deep, localized basins for CA6 and CA3, comparable to the reference inhibitor DJM, indicating favorable thermodynamic stabilization and a high probability of sustained inhibition. The agreement between the kinetic (RMSD) and thermodynamic (ΔG profiles) indicators strongly supports the robustness of the multilevel screening strategy. In addition, the overall computational behavior of the three shortlisted candidates relative to the reference inhibitors is summarized in Figure S8. This comparative analysis integrates the multilevel metrics used throughout the study, including ensemble docking, MD-derived stability, WT-MetaD free-energy descriptors, and multidimensional similarity to each control, providing a compact visualization that highlights the strong convergence between the computational profile of CA1, CA3, and CA6 and that of the potent inhibitors DJM, AHA, and BME. This consolidated view reinforces the internal consistency of the workflow and supports the prioritization of these three compounds for future experimental validation.

Despite its comprehensive computational design, this study is not exempt from limitations. The predicted affinities and stability parameters rely on force-field accuracy, the quality of the electron microscopy template, and the sampling times accessible in atomistic simulations. Moreover, while the WT-MetaD approach provides reliable relative free-energy estimates, absolute binding free energies would benefit from complementary enhanced-sampling or alchemical methods. The absence of experimental validation remains an inherent limitation but also represents the next logical step toward confirming the inhibitory activity, metal coordination, and cellular efficacy of the identified triazole candidates. Finally, a targeted ADMT evaluation of the three shortlisted candidates (Table S8) confirmed that all molecules comply with major drug-likeness rules and exhibit physicochemical and permeability profiles compatible with small-molecule UI. Although ADMETlab flagged potential hepatotoxicity- or genotoxicity-related liabilities in some candidates, these predictions primarily serve as early-stage alerts and reinforce the need for routine in vitro profiling during the next phase of evaluation.

Importantly, the chemical space analysis demonstrated that the top candidates (CA1, CA3, and CA6) occupy molecular regions closely associated with potent UI, particularly DJM, confirming that the computationally derived hits are chemically coherent within the inhibitor landscape. This convergence between energetic, dynamical, and cheminformatics descriptors underscores the predictive reliability of the multistage workflow and establishes a robust foundation for future in vitro validation and lead optimization.

## Figures and Tables

**Table 1:** Machine learning models selected from our previous study [20] for the classification of UI and nUIs. Each model is defined by the combination of algorithm, feature selection strategy, and bioactivity categorization scheme based on IC_50_.

Model	ML Algorithms	Feature Selection Method	Bioactivity Categorization Schemes
1	Decision tree (DT)	Boruta	5–50 µM
2	eXtreme Gradient Boosting (XGB)	Non-Feature Selection (nFS)	25–20 µM
3	Decision tree (DT)	eXtreme Gradient Boosting (XGB)	5 µM
4	k-nearest neighbor (kNN)	eXtreme Gradient Boosting (XGB)	5–50 µM
5	Random forest (RF)	Boruta	10–50 µM

## Data Availability

The original data supporting the findings of this study are openly available within the electronic Supporting Information. The R-language scripts and data were deposited in GitHub link: https://github.com/DanielBustosG/Integrative-Computational-Approaches-for-Urease-Inhibitor-Discovery--An-Integrated-Machine-Learning (accessed on 23 November 2025).

## Supplementary Materials

The following supporting information can be downloaded at: https://www.mdpi.com/article/10.3390/ijms262311576/s1, Additional results supporting the computational workflow—including pharmacophore assessment, docking analyses, MD-based stability metrics, free-energy reconstructions, chemical-space exploration, and ADMET predictions.
