# Supplementary material for: Integrative Computational Approaches for the Discovery of Triazole-Based Urease Inhibitors: A Machine Learning, Virtual Screening, and Meta-Dynamics Framework
Source: Int J Mol Sci. 2025 Nov 28;26(23):11576. doi: 10.3390/ijms262311576 (PMC12692664; doi:10.3390/ijms262311576)
Supplement: Supplementary file 1 [file ijms-26-11576-s001.zip › ijms-3986045-supplementary.pdf]

International Journal of *Molecular Sciences*

MDPI

Article

Integrative Computational Approaches for the Discovery of Triazole-Based Urease Inhibitors: A Machine Learning, Virtual Screening, and Meta-Dynamics Framework

Sofía E. Ríos-Rozas [1,†], Natalia Morales [1,2,†], Elizabeth Valdés-Muñoz [1,3], Gabriela Urra [1], Camila A. Flores-Morales [1,2], Javier Farías-Abarca [1], Erix W. Hernández-Rodríguez [1], Jonathan M. Palma [4], Manuel I. Osorio [5], Osvaldo Yáñez-Osses [6], Luis Morales-Quintana [7], Reynier Suardíaz [8,*] and Daniel Bustos [1,*]

[1] Laboratorio de Bioinformática y Química Computacional, Departamento de Medicina Traslacional, Facultad de Medicina, Universidad Católica del Maule, Talca 3480094, Chile; javier.farias@alu.ucm.cl (J.F.-A.); ehernandez@ucm.cl (E.W.H.-R.)
[2] Doctorado en Ingeniería, Facultad de Ingeniería, Universidad Católica del Maule, Talca 3480094, Chile
[3] Doctorado en Biotecnología Traslacional, Facultad de Ciencias Agrarias y Forestales, Universidad Católica del Maule, Talca 3480094, Chile
[4] Facultad de Ingeniería, Universidad de Talca, Maule, Curicó 3349001, Chile; jonathan.palma@utalca.cl
[5] Facultad de Odontología, Universidad Andrés Bello, Santiago Chile, Echaurren 237, Santiago 8370133, Chile; manuel.osorio@unab.cl
[6] Centro de Modelación Ambiental y Dinámica de Sistemas (CEMADIS), Facultad de Ingeniería y Negocios, Universidad de Las Américas, Santiago 7500975, Chile; oyanez@udla.cl
[7] Multidisciplinary Agroindustry Research Laboratory, Instituto de Ciencias Biomédicas, Facultad de Ciencias de La Salud, Universidad Autónoma de Chile, Cinco Poniente #1670, Región del Maule, Talca 7500912, Chile; luis.morales@uautonoma.cl
[8] Departamento de Química Física, Facultad de Ciencias Químicas, Universidad Complutense de Madrid, 28040 Madrid, Spain
* Correspondence: reysuard@ucm.es (R.S.); dbustos@ucm.cl (D.B.)
† These authors contributed equally to this work.

Table S1. Evaluation metrics of the four pharmacophore hypotheses generated using triazole-based UI as templates. The table summarizes the BEDROC scores, number of active and inactive molecules, and the active/inactive ratios used to select the optimal pharmacophore model.

Pharmacophore hypothesis	BEDROC Score	Active Molecules	Inactive Molecules	Ratio Active/Inactive
AHRR	0.627447	74	3	24.66
AAAR	0.622203	70	2	35
AHRR	0.475818	65	11	5.90
AARR	0.492758	78	25	3.12

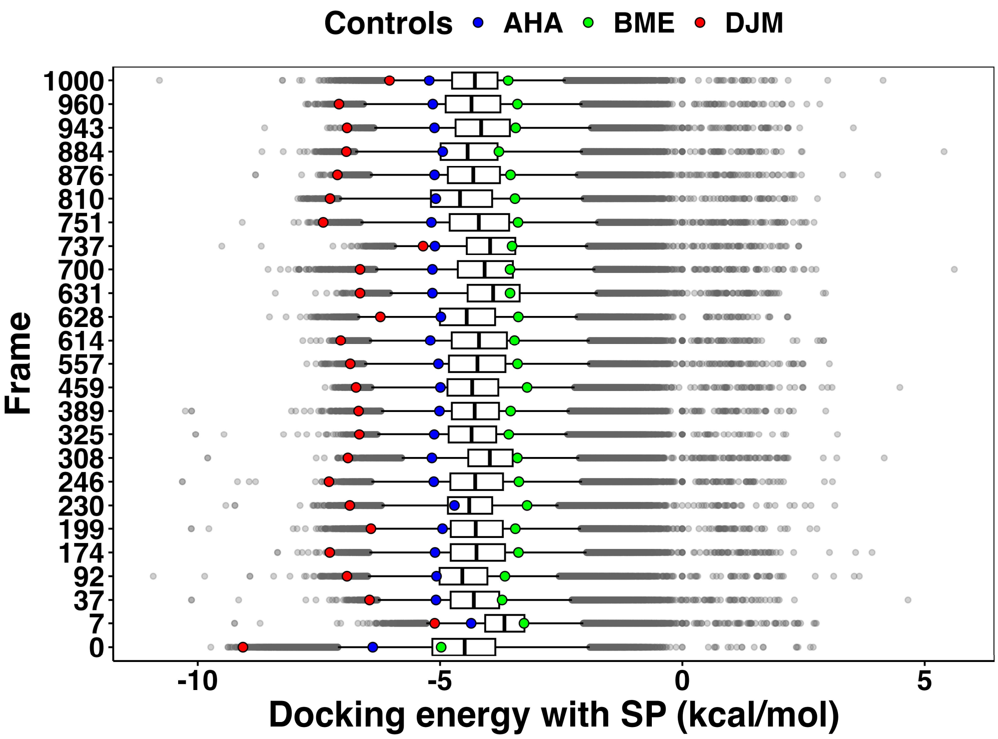

Figure S1. Distribution of docking energies across the 25 conformational frames of HpU obtained from MD simulations. Gray boxplots represent the energy distribution of the 80,530 docked compounds using SP-Glide, while colored points indicate the GM binding energies of the control inhibitors DJM (red), AHA (blue), and BME (green).

Table S2. Statistical summary of GM docking energies (kcal/mol) for the three control inhibitors and the ZINC database subset into the SP stage of Glide.

Molecules	Q1	Median	Q3	Mean	S.D.	# ZINC molecules under the control
BME	-3.719	-3.492	-3.253	-3.522	0.450	66,112
AHA	-5.063	-5.063	-4.911	-5.093	0.422	7,062
DJM	-7.213	-6.915	-6.538	-6.747	0.897	7
ZINC DB subset	-4.651	-4.213	-3.729	-4.152	0.785	

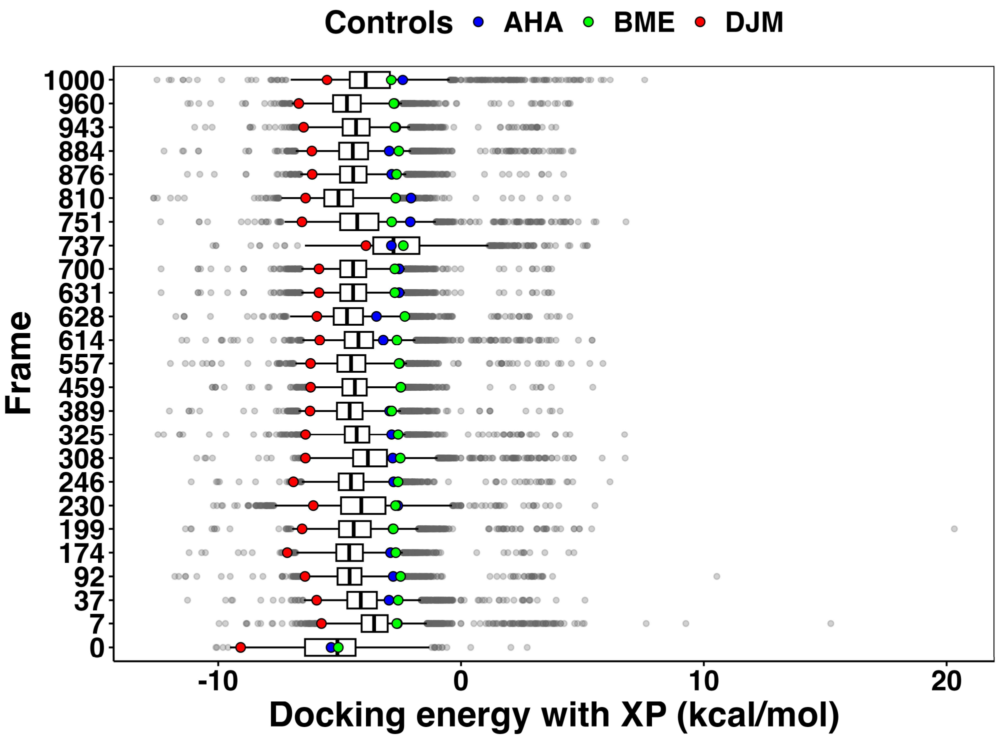

Figure S2. Distribution of docking energies across the 25 conformational frames of HpU obtained from MD simulations. Gray boxplots represent the energy distribution of the 7,062 docked compounds using XP-Glide, while colored points indicate the GM binding energies of the control inhibitors DJM (red), AHA (blue), and BME (green).

Table S3. Statistical summary of GM docking energies (kcal/mol) for the three control inhibitors and the ZINC database subset into the XP stage of Glide.

Molecules	Q1	Median	Q3	Mean	S.D.	# ZINC molecules under the control
BME	-2.881	-2.681	-2.464	-2.715	0.623	6,861
AHA	-2.961	-2.873	-2.622	-2.800	0.612	6,811
DJM	-6.630	-6.274	-5.703	-6.119	1.118	16
ZINC DB subset	-4.534	-4.109	-3.633	-4.06	0.724	

Table S4. The top 7 candidates named CA1 to CA7, with their ZINC code and structure. DJM, AHA and BME are the UI used as controls.

ZINC code	Abbreviation of candidate (CA) molecule	2D representation
ZINC000000947726	CA1	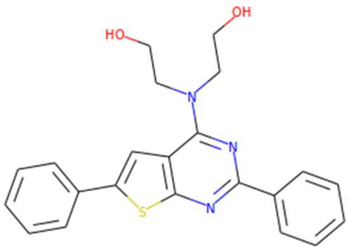
ZINC000003995957	CA2	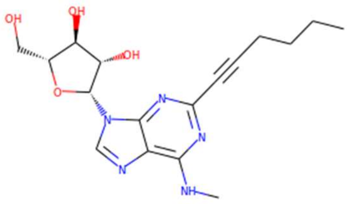
ZINC000005317978	CA3	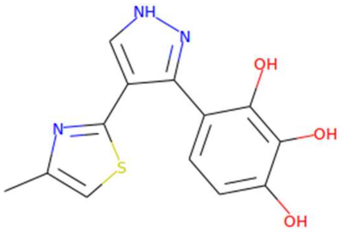
ZINC000026959979	CA4	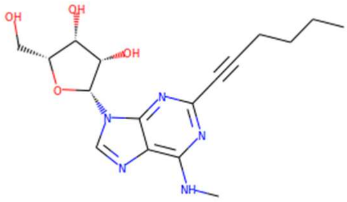
ZINC000067803045	CA5	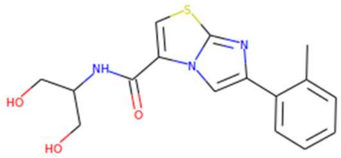
ZINC000067909942	CA6	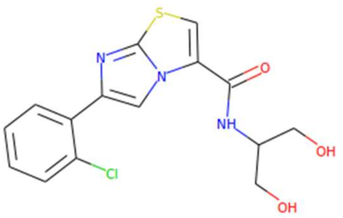

ZINC000091409996	CA7	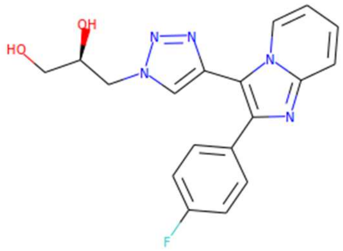
2-{[1-(3,5-dimetilfe-nil)-1H-imidazol-2-il]sulfanil}-N-	DJM	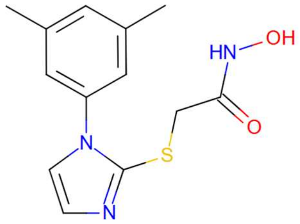
Ácido acetohidroxá-mico	AHA	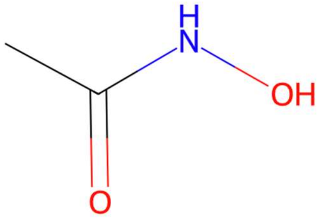
β-mercaptoetanol	BME	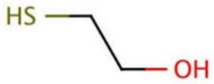

Table S5. Descriptive statistics of ligand RMSD values (Å) computed over the final 20 ns of the MD simulations under unrestrained conditions. The table summarizes quartile (Q1–Q3), mean, and standard deviation (S.D.) values for the control inhibitors and candidate molecules (CA1–CA7).

Molecule	Q1	Median	Q3	Mean	S.D.
BME	0.0482	0.0565	0.0688	0.0585	0.0189
AHA	0.0465	0.0555	0.0722	0.0568	0.0181
DJM	0.5820	0.7270	10.300	0.7990	0.3490
CA1	0.3620	0.4420	0.6240	0.4820	0.1650
CA2	0.6580	0.8020	0.9010	0.7660	0.2240
CA3	0.2320	0.2810	0.3510	0.2910	0.1200
CA4	0.6210	0.8620	0.9560	0.7810	0.2490
CA5	0.6350	0.6720	14.700	0.9450	0.4670
CA6	0.5760	0.6800	0.7480	0.6200	0.1950
CA7	0.9040	0.9580	10.300	0.8980	0.2420

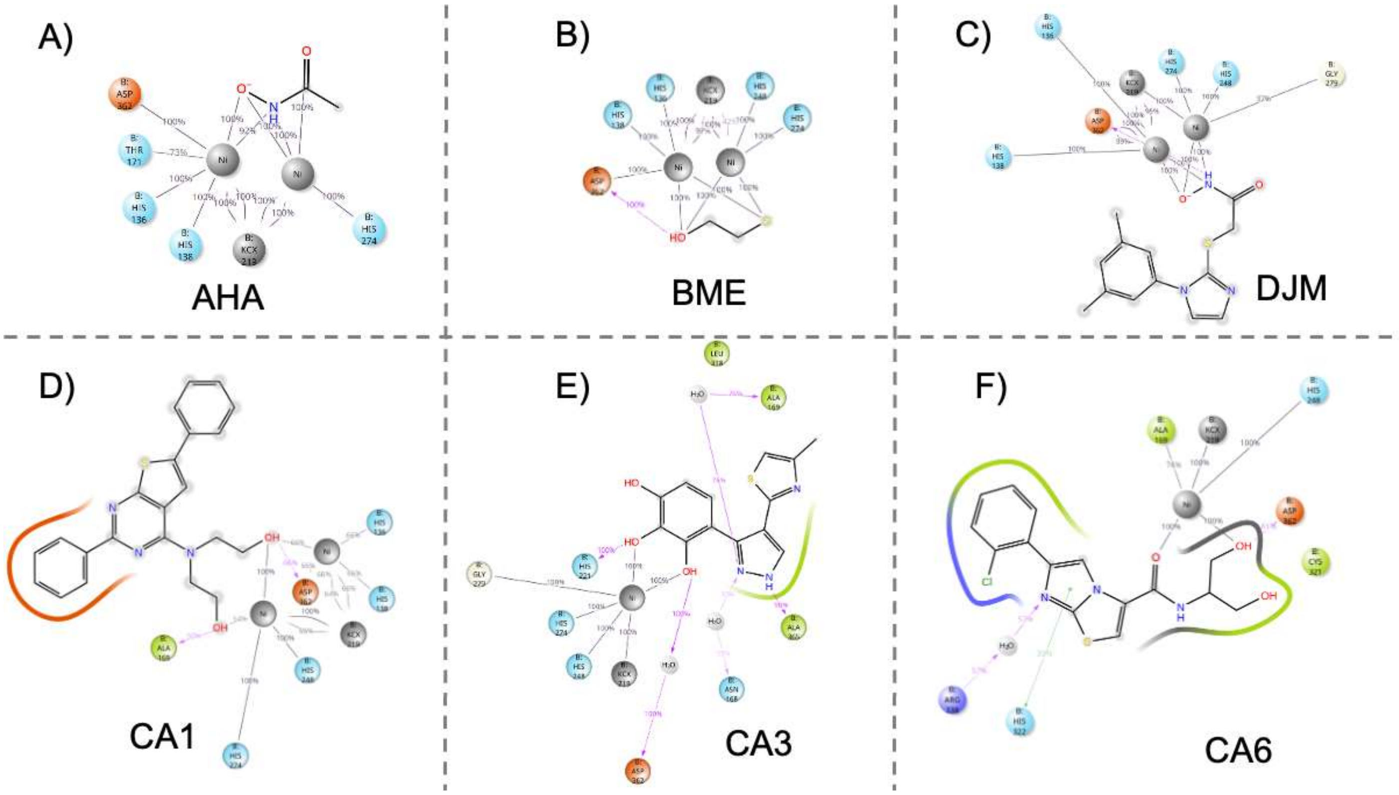

Figure S3. Predominant interactions between HpU and selected ligands during the last 20 ns of unrestrained MD simulations. Panels (A–C) correspond to the reference inhibitors AHA, BME, and DJM, respectively, whereas panels (D–F) show the top three candidate molecules CA1, CA3, and CA6. Nickel ions are represented as gray spheres. Coordination bonds and hydrogen-bond interactions are depicted as purple lines, with percentages indicating their persistence throughout the analyzed trajectory.

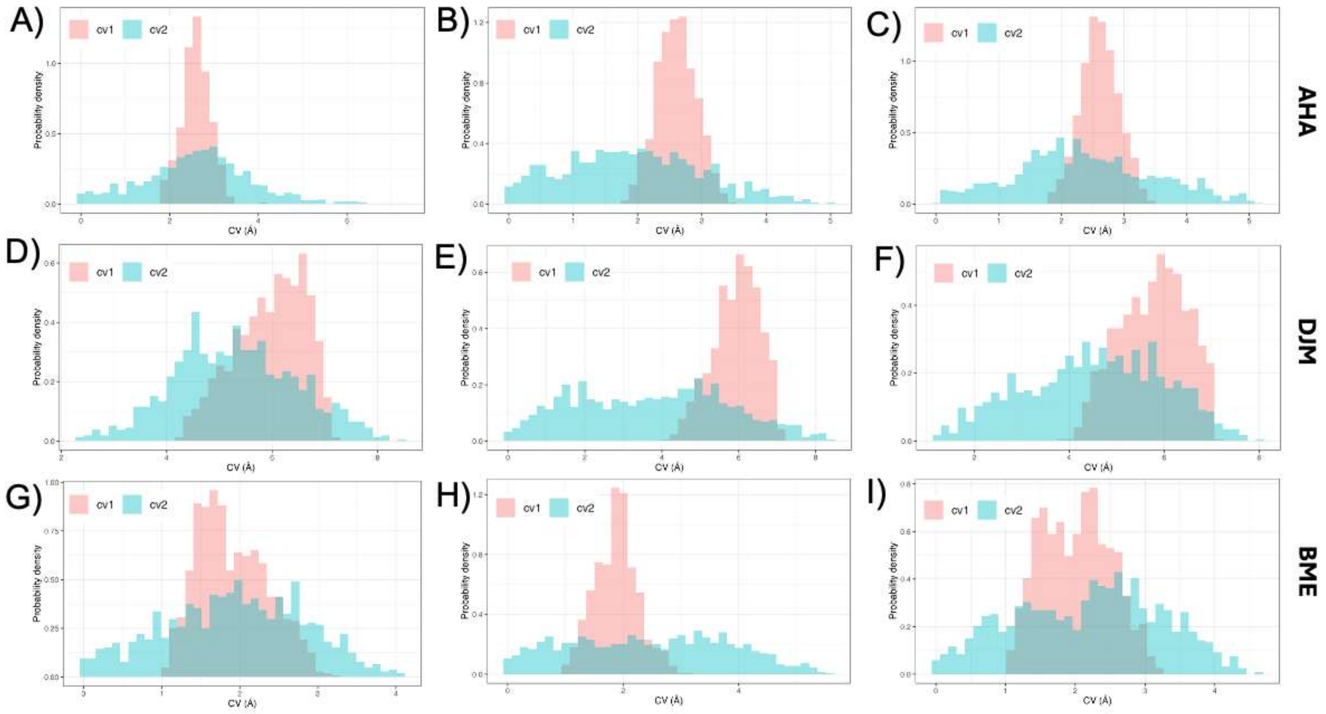

Figure S4. Probability density histograms of collective variables (CV1 and CV2) obtained from WT-MetaD simulations of the control inhibitors AHA (A–C), DJM (D–F), and BME (G–I), corresponding to three independent replicas. CV1 (pink) represents the ligand–Ni^{2+} distance, and CV2 (blue) represents distance between the COM of key active-site residues (KCX219, H274, C321, D362, A365; heavy atoms only) and the COM of ligand heavy atoms.

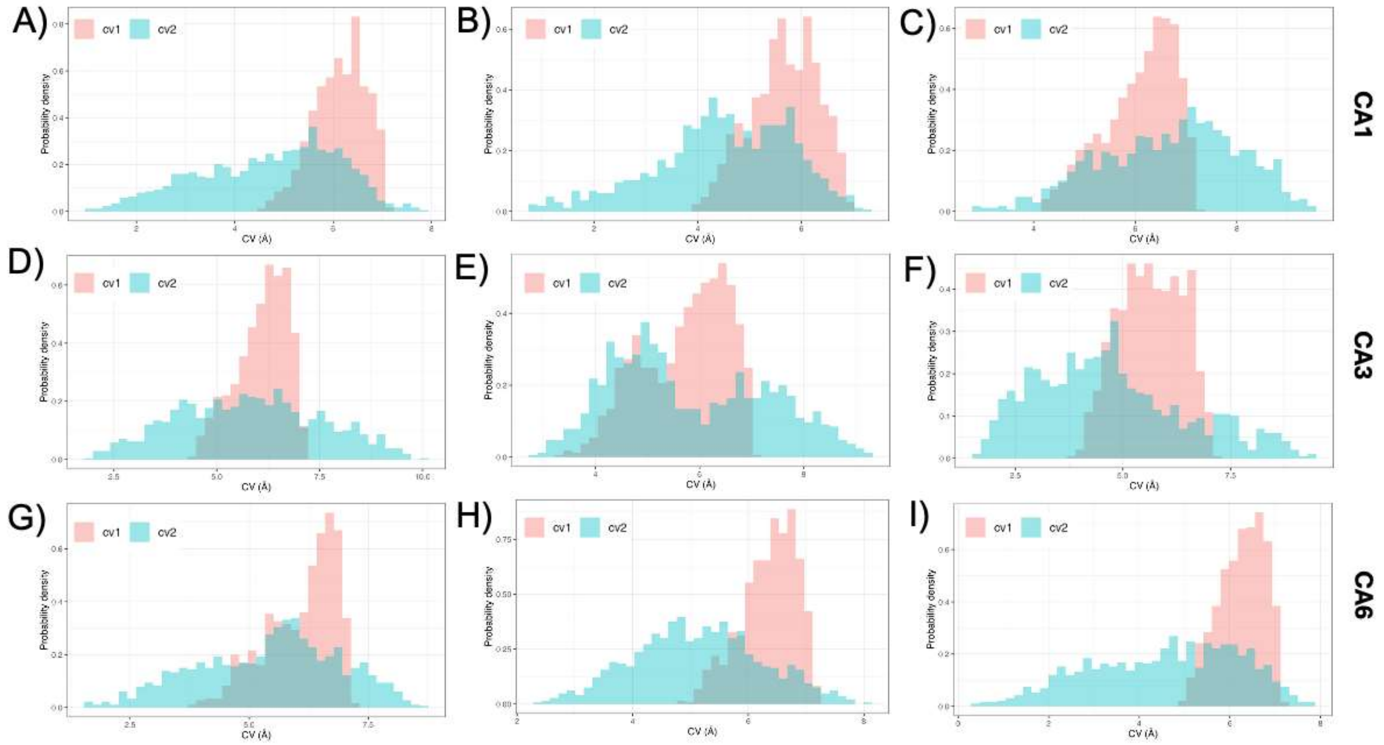

Figure S5. Probability density histograms of collective variables (CV1 and CV2) obtained from WT-MetaD simulations of the top three candidates CA1 (A–C), CA3 (D–F), and CA6 (G–I). Panels correspond to three independent replicas. CV1 (pink) represents the ligand–Ni^{2+} distance, and CV2 (blue) represents distance between the COM of key active-site residues (KCX219, H274, C321, D362, A365; heavy atoms only) and the COM of ligand heavy atoms.

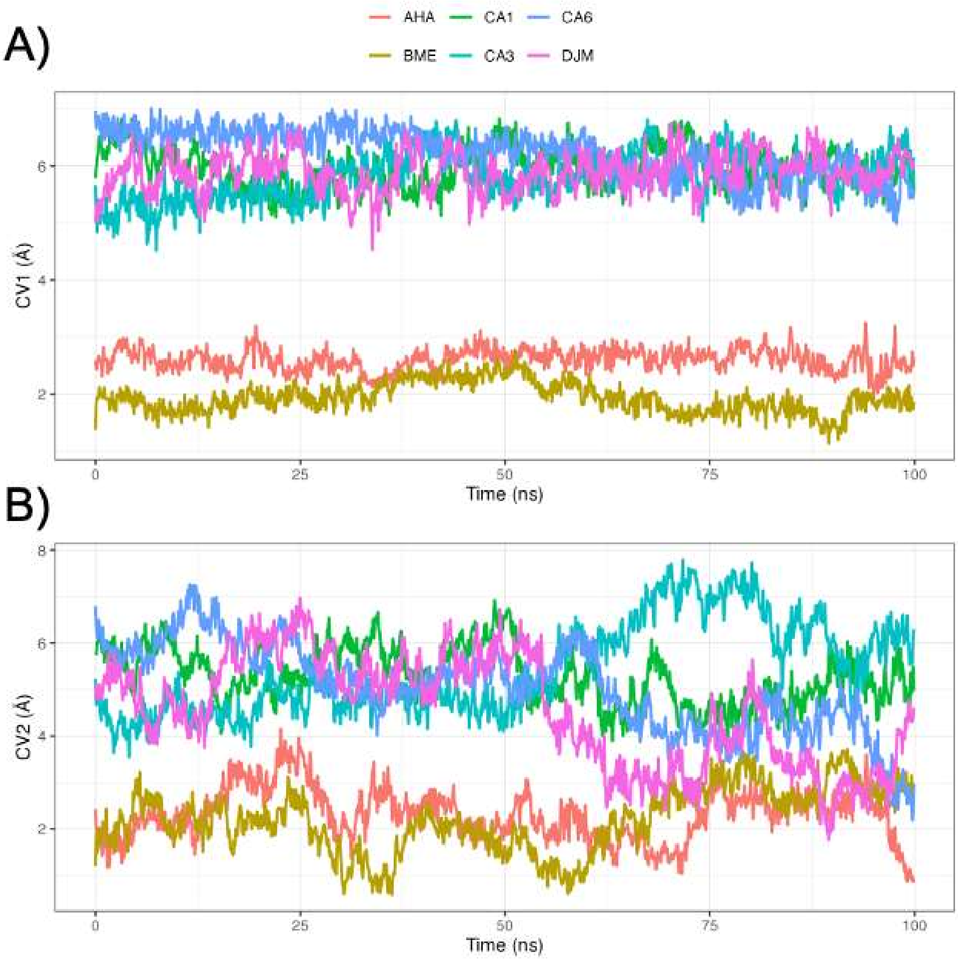

Figure S6. Time evolution of the collective variables (CVs) during WT-MetaD simulations for the control inhibitors (AHA, BME, DJM) and the selected candidates (CA1, CA3, CA6). A) CV1 represents the distance between the center of mass (COM) of ligand heavy atoms and the Ni^{2+} cluster. B) CV2 corresponds to the distance between the COM of key catalytic residues (KCX219, H274, C321, D362, A365; heavy atoms only) and the COM of the ligand. Each trace represents the replicate-averaged trajectory over 100 ns.

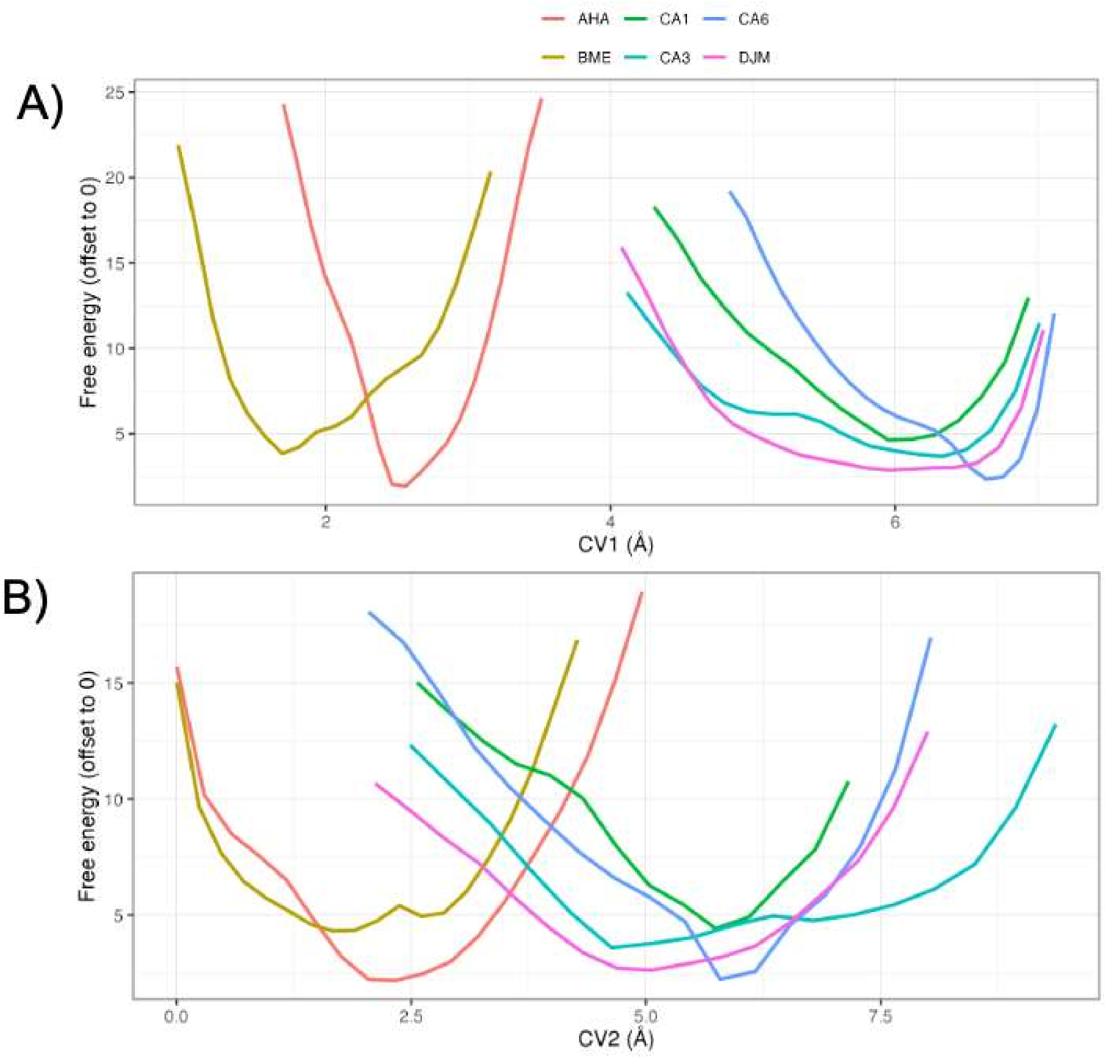

Figure S7. One-dimensional free-energy profiles for all ligands projected along (A) CV1 and (B) CV2 from the WT-MetaD simulations. The minima positions and well depths correspond to the most stable conformational states along each collective variable. DJM, CA6, and CA3 show the lowest energy basins, indicating strong and persistent binding, while AHA and BME display shallow wells typical of weak, metal-adjacent interactions.

Table S6. Estimated computation times per stage. The following table summarizes the approximate wall-times requirements for each major step of our computational workflow. These values are provided to assist readers in reproducing or adapting this multistage pipeline for related drug-discovery applications.

Stage	Approximate Computational Time	Notes
Physicochemical filtering, chemical-space analysis, and ML classification	< 2 hours total	Vectorized operations; feasible even on low-resource machines.
PBVS	~48–72 hours	Throughput ~50,000–100,000 molecules/hour; screening of ~4.9 M molecules on a 48-core CPU workstation.
Ensemble Docking – SP Glide	~100 hours total	~4 hours per *Hp*U conformational frame × 25 frames.
Ensemble Docking – XP Glide	~150 hours total	~6 hours per frame × 25 frames.
QPLD	~1 hour per ligand (~7 hours for 7 final candidates)	Includes QM partial-charge calculation and XP redocking.
All-atom MD simulations (100 ns)	~12 hours per complex	Single NVIDIA RTX 3080 GPU per simulation.
WT-MetaD (100 ns × 3 replicas)	~36 hours per ligand	~12 hours per replica on one NVIDIA RTX 3080 GPU.

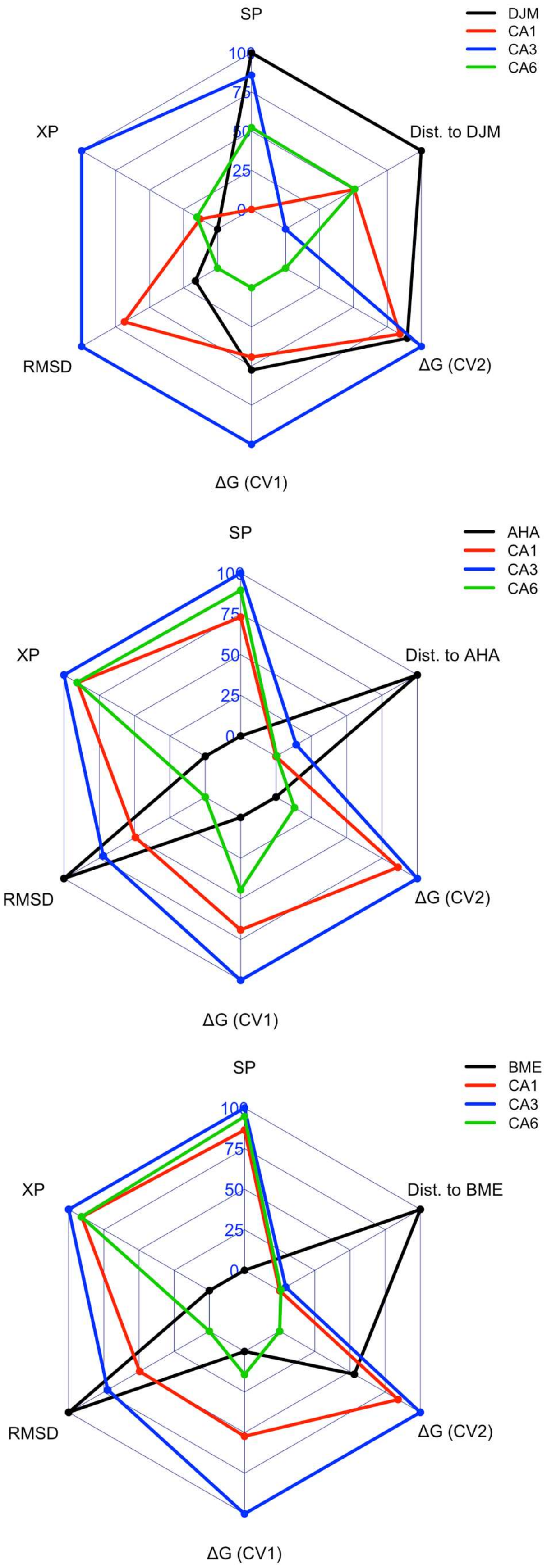
SP
DJM
CA1
CA3
CA6
Dist. to DJM
XP
ΔG (CV2)
RMSD
ΔG (CV1)
0
25
50
75
100
SP
AHA
CA1
CA3
CA6
Dist. to AHA
XP
ΔG (CV2)
RMSD
ΔG (CV1)
SP
BME
CA1
CA3
CA6
Dist. to BME
XP
ΔG (CV2)
RMSD
ΔG (CV1)

Figure S8. Normalized radar plots comparing the computational profiles of the three reference inhibitors (DJM, AHA, and BME) with the top-ranked candidates (CA1, CA3, and CA6). Each axis represents one of the multilevel metrics used throughout the VS workflow: SP docking score, XP docking score, RMSD from MD simulations, WT-MetaD-derived free-energy descriptors (ΔG for CV1 and CV2), and multidimensional cheminformatics distance to each control inhibitor. Higher values indicate better relative performance after normalization to the [0–1] range. The three panels display comparisons against DJM (left), AHA (center), and BME (right).

Table S7. Nearest-neighbor analysis in UMAP chemical space for the three top-ranked triazole candidates (CA1, CA3, and CA6). For each candidate, the five closest experimental HpU inhibitors (based on UMAP Euclidean distance) are listed together with their corresponding SMILES strings, the experimentally reported IC_{50} values (μM) and the mean IC_{50} value foreach candidate.

Candidate reference	Neighbor SMILES	IC_{50} (μM)	Mean IC_{50} (μM)
CA1	COc1ccc(C2COc3cc(OC)ccc3C2O)cc1	3867	
CA1	COCCCOc1ccnc(CS(=O)c2nc3ccccc3[nH]2)c1C	0.29	
CA1	COc1ccc(-c2coc3cc(OC)ccc3c2=O)cc1	3527	2469.46
CA1	O=C1OCC(O)=C1c1ccccc1Cl	100	
CA1	COc1ccc(C2CC(O)c3c(OC)c(OC)c(OC)c(OC)c3O2)cc1	4853	
CA6	O=C(Nc1ccccc1)c1ccccc1[Se][Se]c1ccccc1C(=O)Nc1ccccc1	6.7	
CA6	COc1ccc(-c2nc(C(=O)NC3CCCCC3)cs2)cc1	1.82	
CA6	O=C(O)CNC(=O)c1ccccc1[Se][Se]c1ccccc1C(=O)NCC(=O)O	121.5	34.28
CA6	O=C(O)CCCNC(=O)c1ccccc1[Se][Se]c1ccccc1C(=O)NCCCC(=O)O	35.4	
CA6	COC(=O)CCCNC(=O)c1ccccc1[Se][Se]c1ccccc1C(=O)NCCCC(=O)OC	5.992	
CA3	O=C(O)C(C[P+](=O)O)c1ccc(O)c(O)c1	34.65	
CA3	CN(O)C(=O)CC(O)c1ccc(OCc2ccccc2)c(OCc2ccccc2)c1	376	
CA3	COc1cc(C(O)CC(=O)N(C)O)ccc1OCc1ccccc1	258	152.45
CA3	Oc1cc(O)c2c(c1)OC(c1ccc(O)c(O)c1)C=C2	4.42	
CA3	COc1cccc(C(O)CC(=O)N(C)O)c1	89.2	

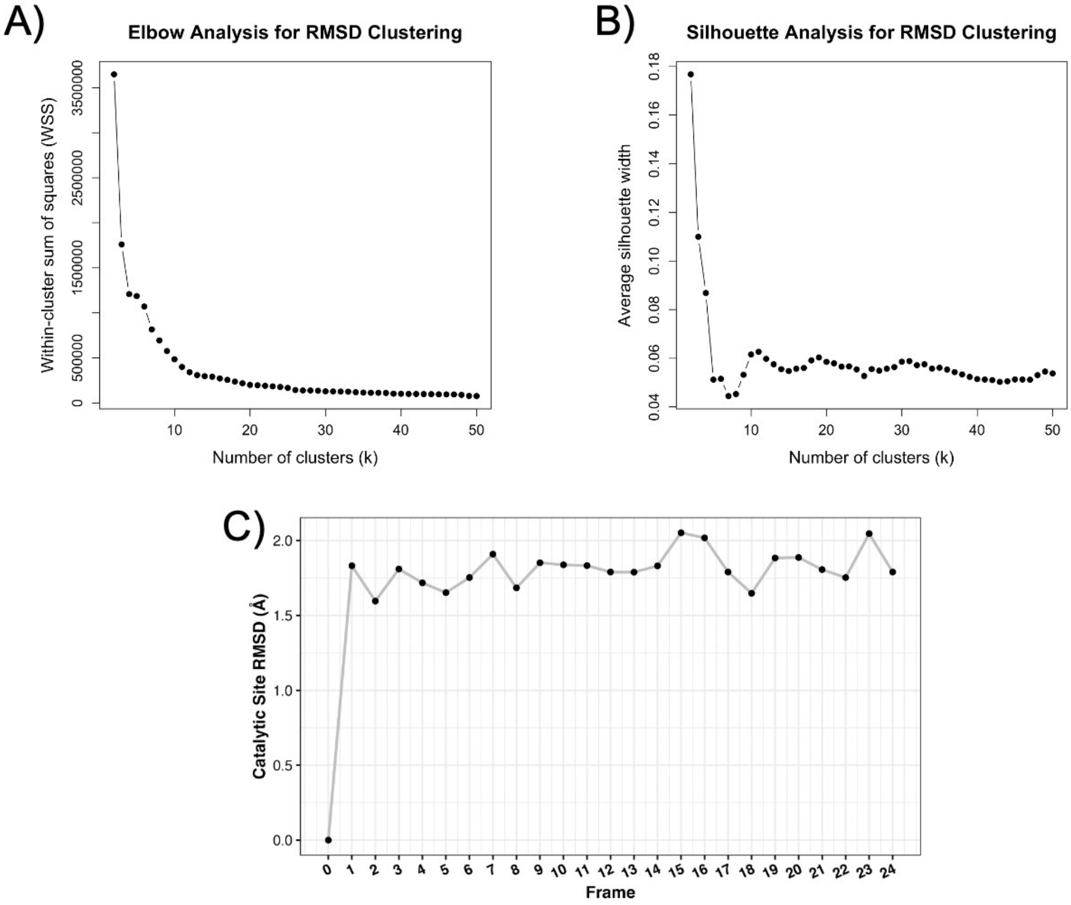

Figure S9. Clustering diagnostics and catalytic-site variability for the 2 μs MD trajectory of *Hp*U. (A) Elbow analysis showing the within-cluster sum of squares (WSS) as a function of the number of clusters (*k*). A sharp decrease is observed up to *k* ≈ 10, followed by a broad plateau extending through k = 50, indicating that cluster partitions above ~10 provide only marginal reductions in structural heterogeneity. (B) Average silhouette width for k = 2–50, illustrating that silhouette values stabilize for *k* ≥ 10 with only minor fluctuations across the range. Together with panel A, this suggests the existence of a wide region of clustering stability rather than a single sharply optimal *k*. (C) Catalytic-site RMSD of the 25 representative frames selected for ensemble docking. The centroid conformations span the full range of fluctuations observed during the trajectory (≈1.6–2.1 Å), confirming that the chosen 25 clusters capture the major microstates explored by the catalytic pocket.

Table S8. Predicted ADMET profiles of the top three candidate UI (CA1, CA3, and CA6) generated using ADMETlab 3.0. The table summarizes key physicochemical, absorption, distribution, metabolism, and toxicity descriptors relevant to early-stage assessment of drug-likeness and safety. All three candidates satisfy major oral drug-like rules and exhibit physicochemical properties consistent with small-molecule inhibitors (MW 289–391 g/mol, TPSA 69–102 Å², logP 2.2–3.8, ≤7 rotatable bonds). Predicted permeability (Caco-2 and MDCK) suggests moderate absorption, while low BBB penetration probabilities align with a gastric-lumen site of action. None of the molecules are predicted as hERG blockers. ADMETlab flags potential liabilitie, including moderate to high probabilities of hepatotoxicity, DILI, and genotoxicity, which are now acknowledged as early-stage risk signals that require confirmation in future *in vitro* assays.

Candidate	Physicochemical Property	Value	Comment
CA1	MW	391.14	Contain hydrogen atoms. Optimal: 100~600
CA3		289.05	
CA6		351.04	
CA1	TPSA	69.48	Topological Polar Surface Area. Optimal: 0~140
CA3		102.26	
CA6		86.86	
CA1	LogP	3.784	The logarithm of the n-octanol/water distribution coefficients at pH=7.4.
CA3		2.203	
CA6		2.383	
CA1	LogS	-4.426	The logarithm of aqueous solubility value
CA3		-2.832	
CA6		-3.797	
CA1	Rotatable bonds	7	Number of rotatable bonds. Optimal:0~11
CA3		2	
CA6		6	

Candidate	Absorption Property	Value	Comment
CA1	Caco-2	-4.950	Optimal: higher than -5.15 Log unit
CA3		-5.239	
CA6		-5.221	
CA1	MDCK	-4.780	low permeability: < 2 × 10-6 cm/s
CA3		-4.816	medium permeability: 2-20 × 10-6 cm/s
CA6		-4.224	high passive permeability: > 20 × 10-6 cm/s
CA1	HIA	0.000	Human Intestinal Absorption
CA3		0.007	Category 1: HIA+(HIA < 30%);
			Category 0: HIA-(HIA >= 30%);
CA6		0.021	The output value is the probability of being HIA+
CA1	P-gp substrate	0.009	Category 1: substrate;
CA3		0.009	Category 0: Non-substrate;
CA6		0.054	The output value is the probability of being Pgp-substrate
CA1	P-gp inhibitor	0.764	Category 1: Inhibitor;
CA3		0.002	Category 0: Non-inhibitor;
CA6		0.202	The output value is the probability of being Pgp-inhibitor

Candidate	Distribution Property	Value	Comment
CA1	BBB	0.664	Blood-Brain Barrier Penetration
CA3		0.363	Category 1: BBB+; Category 0: BBB-;
CA6		0.330	The output value is the probability of being BBB+
CA1	PPB	97.715	Plasma Protein Binding
CA3		96.555	Optimal: < 90%.
CA6		93.014	Drugs with high protein-bound may have a low therapeutic index.
CA1	VDss	0.403	Volume Distribution
CA3		-0.365	Optimal: 0.04-20L/kg
CA6		0.025	

Candidate	Metabolism Property	Value	Comment
CA1	CYP1A2 inhibitor	0.648	Category 1: Inhibitor; Category 0: Non-inhibitor; The output value is the probability of being inhibitor.
CA3		0.999	
CA6		0.015	
CA1	CYP2C9 inhibitor	0.362	
CA3		0.732	
CA6		0.045	
CA1	CYP2C19 inhibitor	0.002	
CA3		0.527	
CA6		0.004	
CA1	CYP3A4 inhibitor	0.006	
CA3		0.983	
CA6		0.000	
CA1	CYP2C8 inhibitor	0.809	
CA3		1.000	
CA6		0.799	

Candidate	Toxicity Property	Value	Comment
CA1	hERG Blockers	0.245	Molecules with IC50 ≤10μM or ≥50% inhibition at 10 μM were classified as hERG+ (Category 1), While molecules with IC50 >10μM or < 50% inhibition at 10μM were classified as hERG - (Category 0). The output value is the probability of being hERG+, within the range of 0 to 1.
CA3		0.077	
CA6		0.017	
CA1	DILI	0.916	Drug Induced Liver Injury. Category 1: drugs with a high risk of DILI; Category 0: drugs with no risk of DILI. The output value is the probability of being toxic.
CA3		0.940	
CA6		0.583	
CA1	Human Hepatotoxicity	0.886	Human Hepatotoxicity Category 1: H-HT positive(+); Category 0: H-HT negative(-); The output value is the probability of being toxic
CA3		0.570	
CA6		0.544	
CA1	AMES Muta genicity	0.705	AMES Toxicity Category 1: Ames positive(+); Category 0: Ames negative(-); The output value is the probability of being toxic.
CA3		0.674	
CA6		0.381	
CA1	Genotoxicity	0.995	Category 0: non-Genotoxicity (-); Category 1: Genotoxicity (+). The output value is the probability of being ototoxicity (+), within the range of 0 to 1.
CA3		0.990	
CA6		0.943	

Disclaimer/Publisher's Note: The statements, opinions and data contained in all publications are solely those of the individual author(s) and contributor(s) and not of MDPI and/or the editor(s). MDPI and/or the editor(s) disclaim responsibility for any injury to people or property resulting from any ideas, methods, instructions or products referred to in the content.
